# Single-cell RNA sequencing reveals that myeloid S100A8/A9 is a novel regulator of the transition from adaptive hypertrophy to heart failure after pressure overload

**DOI:** 10.7150/thno.118369

**Published:** 2025-07-28

**Authors:** Wei-Jia Yu, Wen-Xi Jiang, Shu-Jing Liu, Hui-Hua Li, Qiu-Yue Lin

**Affiliations:** Institute of Cardiovascular Diseases, First Affiliated Hospital of Dalian Medical University, Dalian, China.

**Keywords:** S100A8/A9, macrophages, neutrophils, inflammation, cardiac hypertrophy, heart failure

## Abstract

**Rationale:** Infiltration of immune cells into the heart plays a crucial role in the transition from adaptive hypertrophy to heart failure (HF) following chronic pressure overload. However, the key factors in myeloid cells that regulate this process are still not well defined. Here, we studied the functional role of S100A8/A9 in myeloid cells during this transition.

**Methods:** Cardiac hypertrophy and HF models were induced by transverse aortic constriction (TAC) for 1 to 4 weeks. The heterogeneity of CD45^+^ immune cells and the cellular sources of S100A8/A9 were analyzed using published single-cell RNA sequencing datasets. The effects of S100A8/A9 on TAC-induced hypertrophy and HF were verified in S100A9 knockout (KO) and bone marrow (BM)-chimeric mice and in an *in vitro* coculture system.

**Results:** S100A8/A9 levels were significantly increased in HF patients and in TAC-induced HF model mice. Moreover, the TAC-induced transition from adaptive hypertrophy to HF was significantly attenuated in S100A9-KO mice and WT mice transplanted with S100A9-KO BM cells. Mechanistically, TAC-stimulated upregulation of S100A8/A9 in neutrophils induced an early inflammatory response and adaptive hypertrophy through activation of the p38 MAPK/JNK/AP-1 pathway, leading to increased production of IL-1β and chemokines (CCL2 and CCL6). These chemokines promoted the infiltration of CCR2^+^ macrophages to the damaged heart. Therefore, they exhibited upregulation of S100A8/A9, which led to exacerbation of inflammation, cardiac hypertrophy and fibrosis via activation of the NF-κB/NLRP3, AKT/Calcineurin A and TGF-β/Smad2 signaling pathways. Additionally, treating WT mice with the S100A9 inhibitor ABR-238901 prevented TAC-induced cardiac hypertrophy-related dysfunction.

**Conclusion:** The present findings establish an S100A8/A9-related axis between myeloid cells and cardiac cells that drives the pressure overload-induced transition from hypertrophy to HF, suggesting that S100A8/A9 is a promising therapeutic target for this disease.

## Introduction

Heart failure (HF) is a severe clinical syndrome with complex pathogenesis. Chronic cardiac hypertrophic remodeling is an important risk factor for the initiation and progression of HF and death. Despite its complex etiology, cardiac hypertrophic remodeling is often characterized by abnormal alterations in cardiomyocyte size, immune cell subsets, left ventricular (LV) walls, LV chambers, and contractile function in response to injury or overload 1. Initially, cardiac hypertrophy is an adaptive response that maintains normal function after pressure overload. However, prolonged pressure overload results in maladaptive cardiac hypertrophy and HF. Moreover, cardiac myocytes and fibroblasts play central roles in hypertrophic and fibrotic remodeling, and the accumulation of different immune cell subtypes in the injured heart aggravates this process through multiple signaling pathways 1, 2.

Substantial data demonstrate that immune cell recruitment in the heart is an early event during the pathogenesis of cardiac hypertrophy caused by pressure overload 3-5. Although these immune cells infiltrate the heart at different time points following TAC, their effects on cardiac hypertrophy differ from each other. Among the different immune cell subtypes that accumulate in the injured myocardium, infiltrated CCR2^+^ macrophages, neutrophils and T cells have emerged as the key regulators of adverse LV hypertrophy and HF upon pathological stress 1, 3-7. These immune cells not only play roles in phagocytosis and antigen presentation but also produce a range of inflammatory factors and chemokines, including TNF-α, IL-1β and IL-6, which represent the common pathways for cardiomyocyte death, myofibroblast differentiation, and cardiac dysfunction. S100A8/A9 is a calcium-binding heterodimer that is detected mainly in neutrophils and monocytes/macrophages, and it is highly upregulated in these cells upon exposure to various stresses 1. Interestingly, S100A8/A9 has emerged as a new biomarker for HF, sepsis and other diseases 8, 9. Multiple studies have shown that S100A8/A9 plays a vital role in the pathogenesis of inflammatory diseases, including sepsis, myocardial infarction (MI), ischemia/reperfusion, cardiac fibrosis and renal fibrosis; targeting S100A8/A9 effectively improves these diseases and adverse outcomes 10-15. However, the significance of S100A8/A9 in the transition from adaptive to maladaptive cardiac hypertrophy caused by pressure overload has not been completely elucidated.

In recent years, single-cell RNA sequencing (scRNA-seq) has revealed the cellular heterogeneity and composition of immune cells in different organs or tissues after various stresses in an unbiased manner. Researchers have used scRNA-seq analysis to define the cardiac immune composition in a murine HF model induced by pressure overload 3. However, the roles of myeloid cells and their released mediators in regulating the infiltration of myeloid cells into the heart in the early and late phases of pressure overload-induced HF are poorly understood. In the present study, on the basis of scRNA-seq data, we used complementary mouse and coculture models to explore the role of myeloid cells and their released mediators, particularly S100A8/A9, in HF progression. The present results indicate that S100A8/A9 in myeloid cells plays a key role in the transition from adaptive cardiac hypertrophy to HF, suggesting that S100A8/A9 is a potential therapeutic target for the treatment of this disease.

## Methods

### Animals and treatment

Eight-week-old wild-type (WT) C57BL/6J mice and systemic S100A9-knockout (S100A9-KO) mice were obtained from The Jackson Laboratory and Cyagen Biosciences Inc., respectively. For genotyping S100A9-KO mice, gene amplification was carried out using a primer pair specific to the mouse S100A9 gene: Forward, 5'-GTA TAT GTG GAG GGA AGC TGT CTC-3'; Reverse, 5'-GTG AAA GGA GGC AGA AAG GAC ATG-3'. All animals were housed under specific pathogen-free (SPF) conditions at 20-26 °C with a 12-hour light/dark cycle, with free access to standard diet and water. WT mice received daily intraperitoneal injections of the S100A8/A9 inhibitor ABR-238901 (E1134, MedChemExpress) at 30 mg/kg/day for four consecutive weeks. To establish angiotensin (Ang) II-induced pressure overload model, WT mice were subcutaneously infused with saline or Ang II (A107852-25mg, Aladdin) at a dose of 1000 ng/kg/min using osmotic mini-pumps (1007D, Alzet) for 3 and 7 days. The animal experimental protocol was reviewed and approved by the Institutional Animal Care and Use Committee (IACUC) of Dalian Medical University (Ethical Approval Number: AEE20027), in compliance with the U.S. National Institutes of Health Guidelines for the Care and Use of Laboratory Animals (NIH Publication No. 8023).

### Transverse aortic constriction operation

For the transverse aortic constriction (TAC) operation, eight-week-old mice were placed in a supine position on the surgical platform after anesthetizing with 1.5% isoflurane (RWD, R510-22-10). A longitudinal skin incision (0.5-1 cm) was made along the ventral midline of the neck and chest using ophthalmic scissors. The connective tissue and lateral muscles were gently separated from the tracheal midline using ophthalmic forceps, and excise a 5 mm segment of tissue between the clavicle and second rib using fine surgical scissors. The aortic arch was fully exposed by separating thymus and then ligated with 6-0 non-absorbent suture, and the 27-G blunt needle was used to produce 75% aortic constriction. After 1 week and 4 weeks of TAC, adaptive myocardial hypertrophy and maladaptive heart failure (HF) model were successfully established, respectively.

### Bone marrow transplantation and chimeric mouse generation

To obtain bone marrow transplantation (BMT) models, WT and S100A9-KO mice were anesthetized using 3% isoflurane delivered via a precision vaporizer. The tibiae and femora were aseptically isolated from both hind limbs and washed by PBS in a laminar flow hood. Carefully removed the articular ends of each bone to expose the marrow cavity by using sterile surgical scissors. Bone marrow cells were then flushed out using a 1 mL syringe filled with heparinized RPMI-1640 medium (Meilunbio, MA0215) supplemented with 1% penicillin-streptomycin solution (PB180120, Pricella). The cell suspension was passed through a 70 μm cell strainer to obtain a single-cell suspension, and viable cells were counted using trypan blue exclusion.

For the BMT procedure, recipient WT or S100A9-KO mice received lethal irradiation (8.5 Gy) delivered by a cobalt-60 source over 30 min. Within 24 h post-irradiation, 5 × 10^6 bone marrow cells from either WT or S100A9-KO donors were intravenously injected via the tail vein using a 27-gauge needle. Transplanted mice were maintained in SPF conditions with autoclaved food and acidified water (pH 2.5-3.0) supplemented with antibiotics to prevent infections. After a six-week engraftment period to allow complete hematopoietic reconstitution, the successful chimeric mice were then randomly assigned to experimental groups and performed TAC operation for another four-week to construct cardiac hypertrophy and heart failure (HF) model.

### Echocardiography

Mice were anesthetized via inhalation of 1.5% isoflurane, then placed in a supine position on a heated surgical platform (maintained at 37 °C to prevent hypothermia) and secured using medical adhesive tape to minimize motion artifacts during imaging. Under continuous anesthesia with 1% isoflurane, high-frequency ultrasound imaging was performed using a Vevo 1100 High-Resolution Imaging System (Visual Sonics Inc.). M-mode echocardiography performed by using a 30 MHz linear-array transducer. The following key cardiac parameters were analyzed: left ventricular (LV) ejection fraction (EF%), fractional shortening (FS%), LV internal diameter (LVID, mm), LV anterior wall (LVAW, mm) and posterior wall (LVPW, mm).

### Histopathological examination

Freshly isolated mouse heart samples were immediately fixed in 4% paraformaldehyde (PFA, Life-iLab, AC28L112) at 4 °C for 24 h to ensure optimal tissue preservation. Subsequently, hearts were dehydrated and infiltrated with molten paraffin wax or OCT to process into paraffin sections (5 μm) or frozen sections (8 μm), respectively. The paraffin sections were performed histopathological staining, including hematoxylin-eosin (H&E, G1120, Solarbio), Masson's trichrome (G1340, Solarbio) and immunohistochemistry with anti-α-SMA antibody (ARG66381, Arigo, 1:1000) according to the instructions of reagents. The frozen sections were performed histopathological staining of wheat germ agglutinin (WGA, Vector Laboratories), Dihydroethidium (DHE, D7008, Sigma), and immunofluorescence staining with anti-α-SMA (ARG66381, Arigo, 1:1000), Collagen III (COL3A1, 22734-1-AP, Proteintech, 1:200), γ-H2AX (ab81299, Abcam, 1:200), Mac-2 (ARG11061, Arigo, 1:2000), Ly6G (A22270, Abclonal, 1:200), S100A8 (15792-1-AP, Proteintech, 1:100) and S100A9 (A9842, Abclonal, 1:100), and subsequently incubated with horseradish peroxidasecoupled antibodies (Sino Biological Inc., SSA011).

### Enzyme-linked immunosorbent assay

The protein concentration of S100A8/A9 heterodimer in mouse plasma was measured with an enzyme-linked immunosorbent assay kit (EM1620, Wuhan Fine Biotech Co., Ltd.) according to the manufacturer's protocols. Briefly, whole blood was collected from mouse right carotid artery and performed centrifugation at 3000 rpm for 10 min. The separated plasma (100 μL per sample) was added to the 96-well plate that coated with Lrg1 antibody. Following the addition of the substrate and stop solution, absorbance at 450 nm was determined using a microplate reader.

### Western blot

Total protein was obtained from the freshly isolated mouse heart samples or cultured cells by using a kit (Keygenbio, KGP250) supplemented with protease inhibitor (K1007, APExBIO) and phosphatase inhibitor (K1012, APExBIO). The BCA protein assay kit (Life-iLab, AP12L025) was used to measure the total protein concentration of each sample. 20-30 μg total protein was separated by sodium dodecyl sulfate (SDS)-polyacrylamide gel electrophoresis (PAGE), and the separated protein was transferred onto polyvinylidene difluoride (PVDF) membranes (ISEQ00010, Millipore). Membrane was incubated with the primary antibodies against S100A8 (15792-1-AP, Proteintech), S100A9 (A9842, Abclonal), NLRP3 (ET1610-93, HUABIO), p-p38 (ARG51850, Arigo), p38 (8690, CST), p-JNK (ARG51807, Arigo), JNK (66210-1-lg, Proteintech), p-c-Jun (AF3095, Affinity), c-Jun (AF6090, Affinity), p-c-Fos (AF3053, Affinity), c-Fos (AF5354, Affinity), p-p65 (bs-0982R, Bioss), p65 (ARG65677, Arigo), p-AKT (4060, CST), AKT (4691, CST), Calcineurin A (CaNA, 2614, CST), TGF-β (WL02998, Wanleibio), p-Smad2 (AP1342, Abclonal), Smad2 (12570-1-AP, Proteintech) and GAPDH (ARG10112, Arigo) at 4 ℃ overnight. The corresponding secondary antibodies (92632210, 92632211, LICOR) were applied and the target bands were displayed through the Odyssey® DLx imaging system.

### Real-time fluorescence quantitative polymerase chain reaction

For the real-time fluorescence quantitative polymerase chain reaction (RT-qPCR) analysis, total RNA was obtained from the freshly isolated mouse heart samples or cultured cells by Trizol method. 1 μg RNA was synthesized the signal-stranded cDNA by PrimeScript RT reagent kit (Yeasen, 11141ES60). qPCR was conducted by using Taq SYBR^®^ Green qPCR Premix (EG20117M, Yugong Biotech) on an Applied Biosystems 7500 Fast system (ABI, USA). The primer sequences used in current study were list in the following table.

### Isolation and treatment of primary mouse BM neutrophils

Following cervical dislocation and surface sterilization in 75% ethanol, mouse femurs and tibias were isolated by skin incision, with attached muscles removed using curved scissors and residual tissues cleared with gauze. Bones were immersed in RPMI-1640 (10% FBS, 2 mM EDTA), and marrow was flushed with a 1ml syringe, then filtered through a 200 μm sieve. After centrifugation (500 g, 5 min, 4 °C), cells were resuspended in PBS, layered onto 55%/65%/80% Percoll gradients, and centrifuged (1200 g, 30 min, 4 °C; low brake). The 65%-80% interface fraction was collected, diluted in PBS, centrifuged (500 g, 5 min), treated with RBC lysis buffer (4 °C, 5 min), and finally resuspended in complete RPMI-1640 (10% FBS, 1% penicillin/streptomycin) for counting. BM neutrophils were stimulated with saline or Ang II (100 nM) for 24 h and proceed with the subsequent experiments.

### Isolation and treatment of primary mouse BM macrophages

The methodology for isolation of primary BM cells from WT and S100A9-KO mice was detailly outlined earlier in the section on bone marrow transplantation and chimeric mouse generation. To generate purified BM-derived macrophages (Mφs), cells were cultured in RPMI-1640 medium (VivaCell, Shanghai, China, C3113-137 0500) supplemented with 10% fetal bovine serum (FBS) and 1% penicillin/streptomycin (PB180120, Pricella), followed by stimulation with 10 ng/ml macrophage-colony stimulating factor (M-CSF) for 3 days. BM Mφs were stimulated with saline or Ang II (100 nM) for 12- and 24-hour and proceed with the subsequent experiments.

### Isolation of primary neonatal rat cardiac myocytes and fibroblasts

Hearts were collected and minced into approximately 1 mm³ fragments using sterile surgical scissors and then subjected to sequential enzymatic digestion with 0.25% trypsin-EDTA (C0201, Beyotime) at 37 °C with gentle agitation. The supernatant containing dissociated cells was immediately transferred to DMEM/F-12 medium (MA0214, MeilunBio) supplemented with 10% FBS (Life-iLab) and 1% penicillin/streptomycin (PB180120, Pricella) to neutralize the enzymatic activity. Cell suspensions were filtered, centrifuged, resuspended in complete DMEM/F-12 medium, and plated onto 100 mm culture dishes for differential adhesion for 90 min at 37 °C in a humidified 5% CO₂ atmosphere. The adherent cardiac fibroblasts (NRCFs) were maintained in DMEM medium (VivaCell, C3113-0500) supplemented with 10% FBS. The non-adherent cardiomyocytes (NRCMs) were collected and resuspended in complete DMEM/F-12 medium. NRCMs were allowed to adhere for 16-20 h under standard culture conditions. Prior to experimental treatments, both NRCMs and NRCFs were synchronized by culturing in serum-free medium for 24 h being used for subsequent experiments.

### Coculture of primary BM Mφs and NRCMs or NRCFs

A transwell-based coculture system was established to investigate the direct effects of BM Mφs on primary NRCMs and NRCFs. Briefly, NRCMs or NRCFs were plated in the lower chamber of a 24-well transwell plate and BM Mφs from either WT or S100A9-KO mice were seeded in the upper chamber. After 12 h of attachment, Mφs were stimulated with saline or Ang II (100 nM), cocultures were maintained for 24 h under standard conditions (37 °C, 5% CO₂). For hypertrophy assessment: NRCMs were performed immunofluorescence staining against anti-α-actinin (ab9465, Abcam) and immunoblotting analysis of p-AKT (4060, CST), AKT (4691, CST) and CaNA (CaNA, 2614, CST). For myofibroblast differentiation: NRCFs were performed α-SMA (ARG66381, Arigo) immunofluorescence staining and immunoblotting analysis of TGF-β (WL02998, Wanleibio), p-Smad2 (AP1342, Abclonal) and Smad2 (12570-1-AP, Proteintech).

### Collection and analysis of clinical patient plasma samples

A total of 262 participants were enrolled from January to December 2022, including 143 patients with hypertension-induced heart failure (HF) and 119 healthy controls. All participants underwent comprehensive evaluation, including physical examination (blood pressure, heart/lung auscultation), echocardiography, laboratory tests (complete blood count, renal/hepatic function, electrolytes), and BNP quantification. Inclusion criteria (HF group) include symptomatic hypertension-induced HF (≥6 months duration), New York Heart Association (NYHA) functional class II-III, LVEF < 40%, elevated BNP > 35 pg/mL, and no medication changes in the preceding 6 months. Exclusion criteria (control group) include recent acute coronary syndrome (MI/unstable angina within 6 months), comorbidities of active infection, autoimmune disease or malignancy, severe pulmonary or renal dysfunction.

This research protocol received formal ethical approval from the Ethics Committee of First Affiliated Hospital of Dalian Medical University (Approval No. LCKY2016-31) and Beijing Chao-Yang Hospital of Capital Medical University (Approval No. 2022-human-244). The study was conducted in strict accordance with the ethical principles of the Declaration of Helsinki. All participants provided written informed consent prior to study enrollment. Patient confidentiality was protected through data anonymization and secure storage protocols.

Blood samples (2 mL) were collected from the antecubital vein of all participants using K₂EDTA Vacutainer™ tubes (BD Biosciences) for the subsequent ELISA assay. Samples were centrifuged at 3,000 g for 15 min at 4 °C to obtain plasma, and then stored at -80 °C until analysis. Plasma levels of S100A8/A9 heterodimer were determined using commercial ELISA kits of S100A8/A9 (EH4140, FineTest). 100 μL plasma samples were loaded in duplicate onto 96-well plates and performed following the manufacturer's standardized protocol. Optical density was measured at 450 nm using a microplate reader, sample concentrations were calculated against standard curves.

### Single-cell sequence analysis

The single-cell RNA-seq data for this study were obtained from GSE122930, including TAC-model mice at different stages (Sham, 1 week, and 4 weeks). Quality control was performed according to a prior study. R package Seurat (v5.2.1) was used for data integration, cell filtration, normalization, clustering, and t-distributed stochastic neighbor embedding (t-SNE) dimensional reduction. Monole2 (v2.30.1) was used for cell pseudotime analysis. Cell progression genes were defined based on DEGs among Seurat clusters. Gene expression was visualized with violin plots, dot plots, heatmaps, and tSNE plots that were created with the Seurat functions VlnPlot, DotPlot, DoHeatmap, and FeaturePlot. The signature markers of each specific cluster were revealed by FindAllMarkers. Differentially expressed genes (DEGs) between two entities were identified by FindMarkers in Seurat. Gene Ontology (GO) enrichment, Kyoto Encyclopedia of Genes and Genomes (KEGG) and Wiki pathway analyses were performed with the enriched genes that were identified with the FindMarkers function with DEGs with the R package clusterProfiler (v 4.13.0).

### Statistical analysis

All data are presented as mean ± standard deviation (SD). Normality of data distribution was assessed using the Shapiro-Wilk test. For comparisons between two groups, parametric data were analyzed by unpaired Student's t-test, and nonparametric data were evaluated using the Mann-Whitney U test. For multiple group comparisons, normally distributed data with homogeneous variance were analyzed by one-way ANOVA. Data violating normality or homogeneity assumptions were examined using Brown-Forsythe ANOVA (unequal variances) or Welch's ANOVA (unequal sample sizes). Spearman correlation test was used to evaluate the association between S100A8/A9 and plasma BNP in patients with HF, and correlation is significant at the 0.01 level (two tailed). p-value < 0.05 indicates statistical significance.

## Results

### S100A8/A9 expression is upregulated in patients and mice with HF

**Figure 1:**
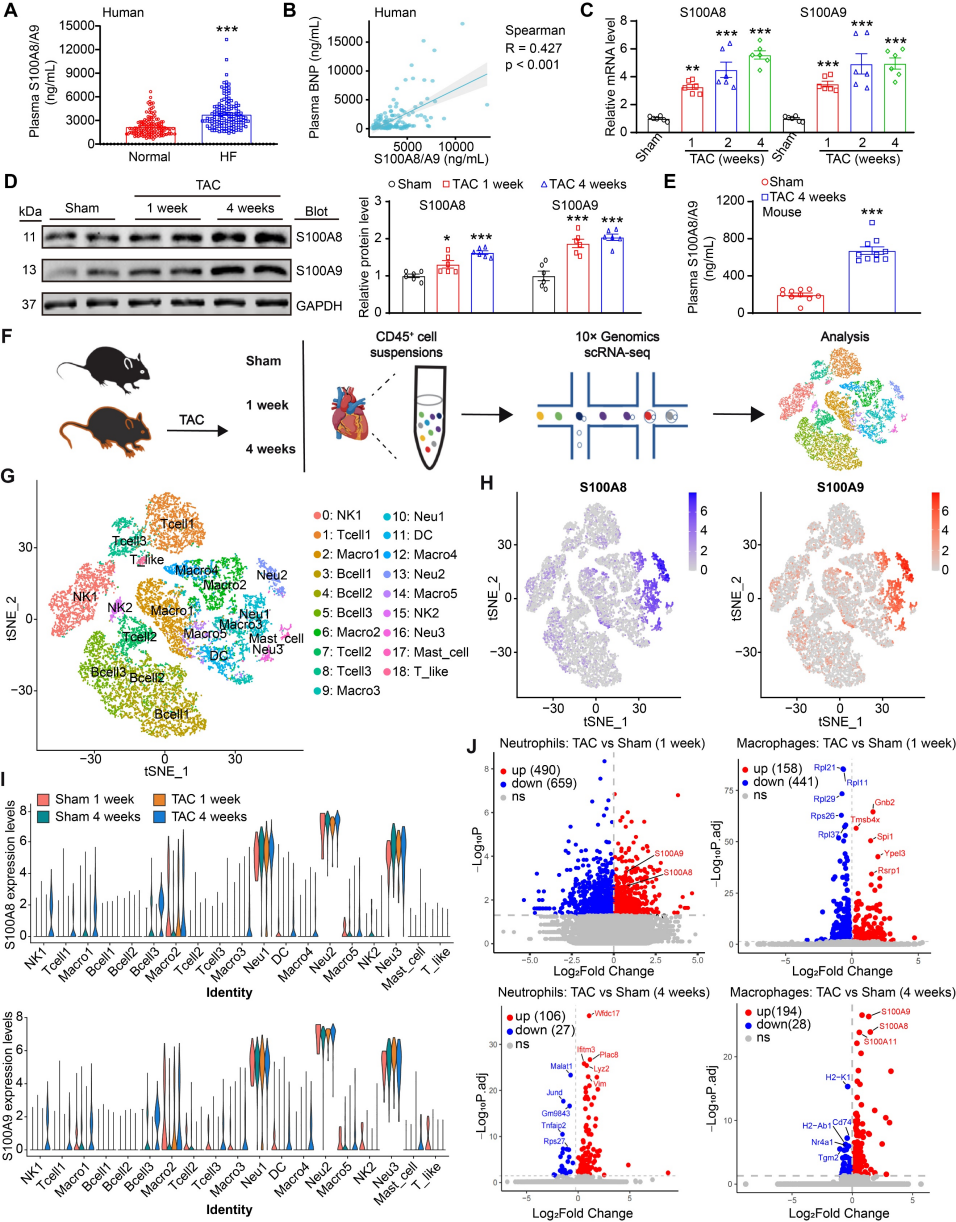
** S100A9 is upregulated during cardiac hypertrophy and heart failure. (A)** Protein concentrations of S100A8/A9 heterodimer in human plasma (n = 119 for normal and n = 143 for HF).** (B)** Association between S100A8/A9 heterodimer and plasma BNP in patients with HF.** (C)** mRNA levels of S100A8/A9 in sham- or TAC-treated hearts (n = 6). **(D)** Representative immunoblots of the S100A8 and S100A9 cardiac signaling proteins and the GAPDH loading control (left). The right panel shows the quantification of these proteins (n = 6). **(E)** Protein levels of S100A8/A9 heterodimer in mouse plasma (n = 10). **(F)** A schematic representation of the single-cell workflow based on a public dataset (GSE122930). Samples were isolated from the hearts of TAC- or sham-operated C57BL6/J male mice 1 or 4 weeks after surgery in duplicate. Hearts were digested, and live CD45^+^ cells were FACS-sorted and loaded for scRNA-seq. **(G)** Two-dimensional t-distributed stochastic neighbor embedding (tSNE) visualization of 17853 cardiac CD45^+^ (immune) cells identified 8 distinct cell types that were classified into 19 clusters after unsupervised clustering. Each point depicts a single cell, which is colored according to cluster designation.** (H)** t-SNE plots showing the expression of the S100A8 and S100A9 genes. The expression levels of the genes are indicated by blue color intensity.** (l)** Violin plots showing the expression levels of the S100A8 and S100A9 genes in all cell types. **(J)** Volcano plots showing differentially expressed genes in neutrophils and macrophages between the TAC (1- and 4-week) and sham groups. The values are presented as the means ± SDs (n = number of humans or animals). *p < 0.05, **p < 0.01 and ***p < 0.001 vs. the normal control or sham group.

To explore whether there is a potential association between plasma S100A8/A9 levels and HF overload, we first obtained plasma samples from healthy subjects (n = 119) and patients with hypertension-induced HF (n = 143). The clinical features of the controls and patients with HF are described in Table S1**.** The HF patients had severe cardiac hypertrophic remodeling and dysfunction, as indicated by a larger LV end-diastolic diameter, a reduced LV ejection fraction (EF%), and higher circulating B-type natriuretic peptide (BNP) levels compared with the healthy subjects (Table S1). Moreover, ELISA analysis revealed a marked increase in S100A8/A9 heterodimer levels (Figure 1A), and a Spearman correlation test revealed a significant association between S100A8/A9 heterodimer levels and plasma BNP in patients with HF (Figure 1B). We subsequently established a mouse model of cardiac hypertrophy and HF induced by pressure overload via TAC surgery. Both qPCR and immunoblot analysis confirmed that S100A8 and S100A9 at both the mRNA and protein levels were increased in the hearts of TAC-treated mice compared with those of the sham group (Figure 1C-D). Furthermore, the protein levels of S100A8/A9 heterodimer in the plasma of TAC-treated mice were greater than those in the heart tissue of sham control mice (Figure 1E). To validate whether cardiac upregulation of S100A8 and S100A9 occurs in other settings of pressure overload, we examined heart tissues from mice infused with angiotensin II (Ang II) for 3 and 7 days, respectively. Immunoblot analysis revealed that the protein levels of S100A8 and S100A9 were greater in Ang II-infused hearts than in saline control hearts (Figure S1A).

### Pressure overload greatly increases S100A8/A9 expression in neutrophils and macrophages during HF

S100A8/A9 is constitutively expressed in myeloid cells, including neutrophils, monocytes/macrophages and dendritic cells, and they are highly upregulated under inflammatory conditions 11. We next determined the dynamic changes in S100A8/A9 in these immune cells of the heart during TAC-induced HF. First, we analyzed total CD45^+^ immune cells to identify cell subpopulations in mouse hearts at weeks 1 and 4 post-TAC, which represent the early and advanced stages of HF, as described previously 3, 16 (Figure 1F). Similar to previous observations 3, the scRNA-seq data revealed 8 distinct cell types among CD45^+^ cells, and these cell types were classified into 19 clusters on the basis of the expression of marker genes via a tSNE map (Figure 1G). The clusters were represented in all 4 conditions (Figure S1B). Interestingly, t-SNE and violin plot analyses indicated that S100A8 and S100A9 were expressed predominantly in neutrophils, followed by macrophages (Figure 1H-I). Compared with those in the control group, the S100A8 and S100A9 expression levels were highly increased in neutrophils at 1 week and in macrophages at 4 weeks after TAC (Figure 1J), indicating that neutrophils and macrophages are the main sources of S100A8/A9 in TAC-treated hearts. Collectively, these data indicated that increased expression of S100A8 and S100A9 in neutrophils may play a major role in the early stage of adaptive cardiac hypertrophy, whereas S100A8 and S100A9 upregulation in macrophages may facilitate cardiac hypertrophy in the late stage of TAC-induced HF.

### S100A8/A9 in neutrophils mediates early inflammatory injury after TAC through the p38 MAPK/JNK/AP-1 signaling pathway

**Figure 2:**
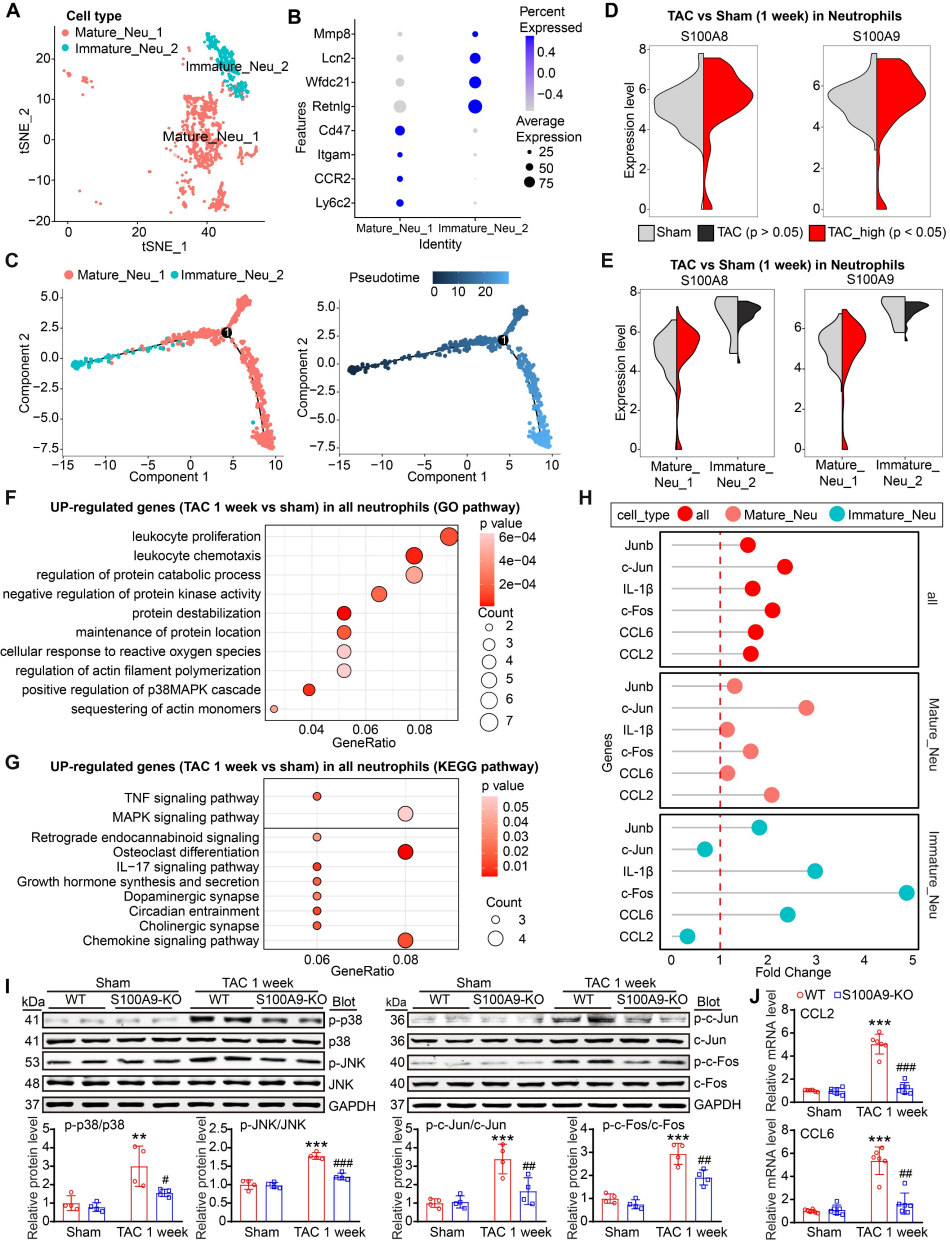
** S100A8/A9 expression is upregulated in cardiac neutrophils at one week post TAC in mice. (A)** t-SNE plot of the two neutrophil subpopulations (Mature_Neu_1 and Immature_Neu_2).** (B)** Dot plots of signature genes confirming the subpopulation identities.** (C)** Monocle analyses showing the ordering of neutrophil cells along pseudotime trajectories. The minute dots in the illustration signify cells, with diverse colors denoting distinct clusters or states.** (D)** Violin plots showing the expression levels of the S100A8 and S100A9 genes in all neutrophils from the TAC and Sham groups at 1 week.** (E)** Violin plots showing the expression levels of the S100A8 and S100A9 genes in the two neutrophil subgroups between the TAC and sham groups at 1 week.** (F)** Top 10 Gene Ontology (GO) pathways associated with genes upregulated in the neutrophils from the TAC group versus those from the Sham group at 1 week. **(G)** The top 10 enriched KEGG pathways associated with the genes whose expression was upregulated in neutrophils from the TAC group versus those from the sham group at 1 week. **(H)** Lollipop plot showing the expression levels of differentially expressed inflammatory factors. **(I)** S100A9-KO and WT mice were subjected to TAC for one week to assess cardiac dysfunction and hypertrophy. Representative immunoblots of the phosphorylated (p)-p38, p38, p-JNK, JNK, p-c-Jun, c-Jun, p-c-Fos, and c-Fos cardiac signaling proteins, as well as the GAPDH loading control (upper). The lower panel shows the quantification of these proteins (n = 4). **(J)** Cardiac qPCR analyses of CCL2 and CCL6 (n = 6). The values are presented as the means ± SDs (n = number of animals). **p < 0.01 and ***p < 0.001 vs. the WT + sham group; ^#^p < 0.05, ^##^p < 0.01 and ^###^p < 0.001 vs. the WT + TAC 1-week group.

Because neutrophils are the first cells recruited to the injury site and the scRNA-seq data revealed that S100A8/A9 expression was upregulated only in neutrophils from the heart at week 1 but not at week 4 post-TAC (Figure 1I-J, Figure S2A), we next investigated which neutrophil cluster is the main source of S100A8/A9 after 1 week of TAC. Two distinct neutrophil clusters, including mature Neu_1 (Cluster 10) and immature Neu_2 (Cluster 13), were identified (Figure 2A). The Neu1 cluster expressed mainly markers of mature neutrophils (Ly6c2, CCR2, Itgam and CD47), whereas the Neu2 cluster exhibited higher expression of immature neutrophil markers (Retnlg, Wfdc21, Lcn2, and Mmp8) (Figure 2B). Pseudotime analysis revealed a transition from immature (Neu_1) to mature (Neu_2) neutrophils at week 1 post-TAC (Figure 2C). Interestingly, compared with those in the control group, both S100A8 and S100A9 expression levels were highly upregulated in the mature Neu_1 subgroup at week 1 post-TAC (Figure 2D-E). Furthermore, GO and KEGG analyses revealed that the upregulated differentially expressed genes (DEGs) were enriched mainly in leukocyte proliferation, chemotaxis, the p38 MAPK pathway, and osteoblast differentiation (Figure 2F-G). These DEGs included S100A8, S100A9, the c-Jun/c-Fos transcription factors (AP-1 complex), chemokines (CCL6 and CCL2) and the IL-1β inflammatory factor, and their expression levels were markedly increased in mature cells (Figure 2H); And several of these genes (IL-1β, c-Fos and CCL6) were also increased in immature cells (Figure 2H). Previous studies have reported that S100A8/A9 increases the expression levels of IL-1β and CCL2 through p38 MAPK/JNK/AP-1 signaling in cancer cells 17-21. Therefore, we assessed these alterations in WT and S100A9-KO mice. After 1 week of TAC, compared with the hearts of WT mice, the hearts of S100A9-KO mice presented significantly lower numbers of inflammatory cells (particularly Mac-2^+^ macrophages, Ly6G^+^S100A8^+^ neutrophils, Ly6G^+^S100A9^+^ neutrophils) and lower levels of proteins (specifically phosphorylated (p)-p38 MAPK, p-JNK, p-c-Jun and p-c-Fos), and mRNAs (specifically CCL2, CCL6 and IL-1β) (Figure 2I-J, Figure S2B-F). Thus, these data suggested that S100A8/A9 activates the p38 MAPK/JNK/AP-1 pathway, which upregulates CCL2 and CCL6 and IL-1β expression, thereby leading to an early inflammatory response induced by TAC.

### S100A9 deficiency improves cardiac contractile function with adaptive hypertrophy after 1 week of TAC

**Figure 3:**
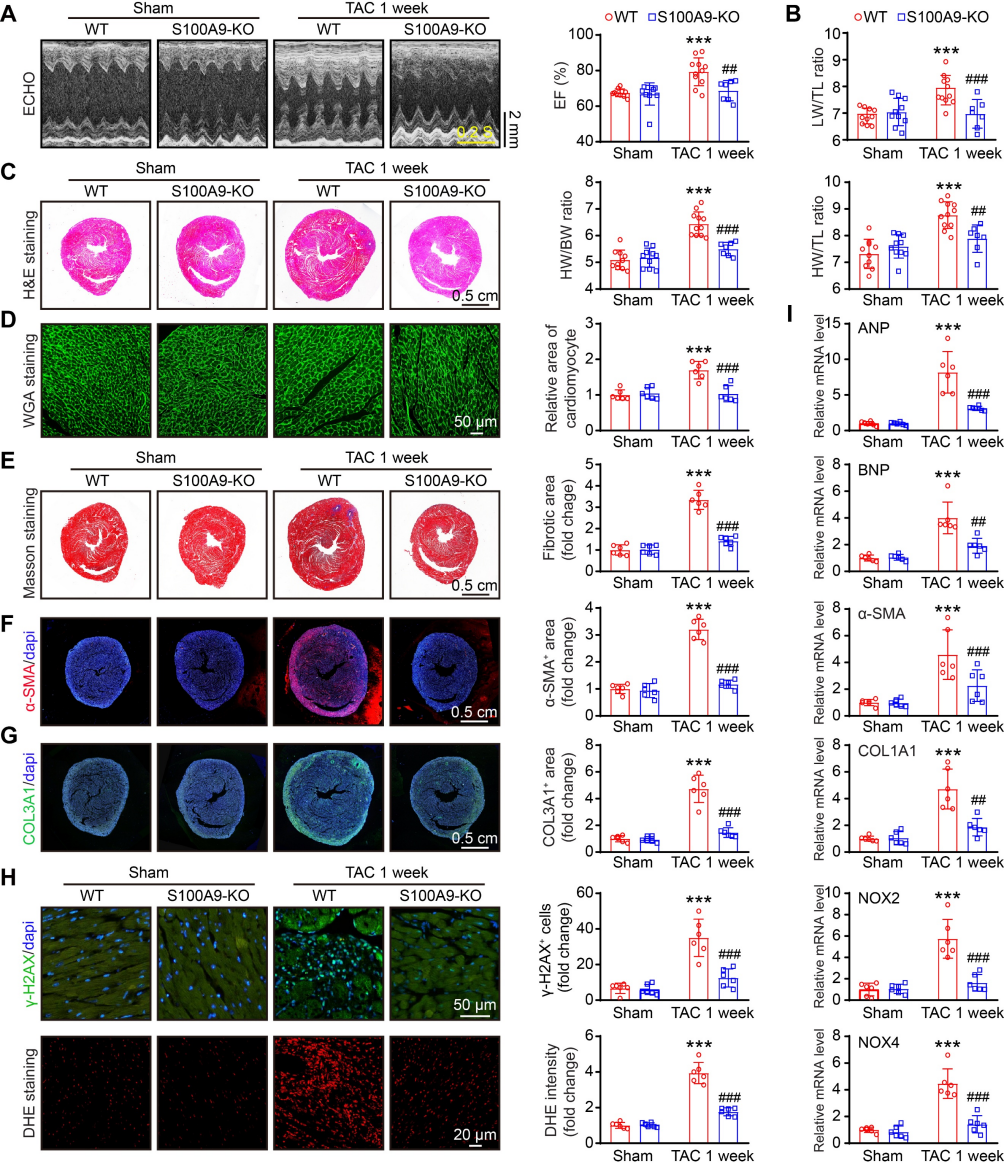
** S100A9 knockout improves cardiac contractility with compensatory hypertrophy at one week post TAC.** S100A9-KO and WT mice were subjected to TAC for one week to assess cardiac dysfunction and remodeling. **(A)** Representative echocardiographic images (left) and quantified ejection fraction (EF%, right, n = 7-11). **(B)** Ratio of the lung weight (LW) to the tibia length (TL, n = 7-11).** (C)** Representative images of H&E-stained cardiac tissue (left) and the ratio of heart weight (HW) to body weight (BW) or to TL (right, n = 7-11). **(D)** Representative images of cardiac tissue subjected to WGA staining (left) and the relative area of cardiomyocytes (right, n = 6). **(E)** Representative images of cardiac tissue subjected to Masson's staining (left) and the quantified fibrotic area (right, n = 6). **(F)** Cardiac immunofluorescence staining with an α-SMA antibody (left, red) and quantification of the results (right, n = 6). **(G)** Cardiac immunofluorescence staining with a COL3A1 antibody (left, green) and quantification of the results (right, n = 6). **(H)** Cardiac immunofluorescence staining with γ-H2AX antibody and quantification of the results (upper, green, n = 6). Cardiac DHE staining and quantification of the results (lower, n = 6).** (I)** Cardiac qPCR analyses of hypertrophic markers (ANP and BNP), fibrosis markers (α-SMA and COL1A1), and oxidative stress markers (NOX2 and NOX4) (n = 6). The values are presented as the means ± SDs (n = number of animals). ***p < 0.001 vs. the WT + sham group; ^##^p < 0.01 and ^###^p < 0.001 vs. the WT + TAC 1-week group.

To explore whether S100A8 or S100A9-mediated inflammation induces cardiac hypertrophy in the early phase of HF, we applied WT and S100A9-KO mice and subjected them to sham or TAC surgery for 1 week. Compared with WT mice, S100A9-KO mice presented no apparent phenotype under basal conditions and exhibited normal cardiac contraction, as evidenced by the EF%, after TAC (Figure 3A). Moreover, the TAC-induced features in WT mice, including lung edema, LV hypertrophy, cellular hypertrophy and a small LV cavity size (as evidenced by the increased ratios of lung/tibial length [LW/TL], heart weight/tibial length [HW/TL] and heart weight/body weight [HW/BW]; myocyte size [H&E and WGA staining]; increased LV anterior wall [AW] and posterior wall [PW] thickness; and reduced LV internal dimension [LVID] in WT mice) were noticeably reversed in S100A9-KO mice (Figure 3B-D, Table S2). Compared with those in WT mice, cardiac fibrosis and oxidative stress (as indicated by the increased fibrotic area [Masson's staining]; the percentages of α-SMA- and collagen III (COL3A1)-positive areas; the number of γ-H2AX-positive cells [a marker of DNA damage]; and the ROS level [DHE staining]) were reduced in S100A9-KO mice (Figure 3E-H). Accordingly, qPCR analysis further confirmed that the mRNA levels of hypertrophic markers (ANP and BNP), fibrosis markers (α-SMA and COL1A1), and oxidative stress markers (NOX2 and NOX4) were noticeably lower in the S100A9-KO mice than in the WT mice following TAC (Figure 3I). There were no statistically significant differences in these parameters between WT and S100A9-KO mice after sham surgery (Figure 3A-I). Collectively, these results suggested that S100A9 induces adaptive cardiac hypertrophy and dysfunction at an early stage of disease.

### S100A8/A9 in CCR2^+^ macrophages amplifies the inflammatory response through the NF-κB and NLRP3 signaling pathways

**Figure 4:**
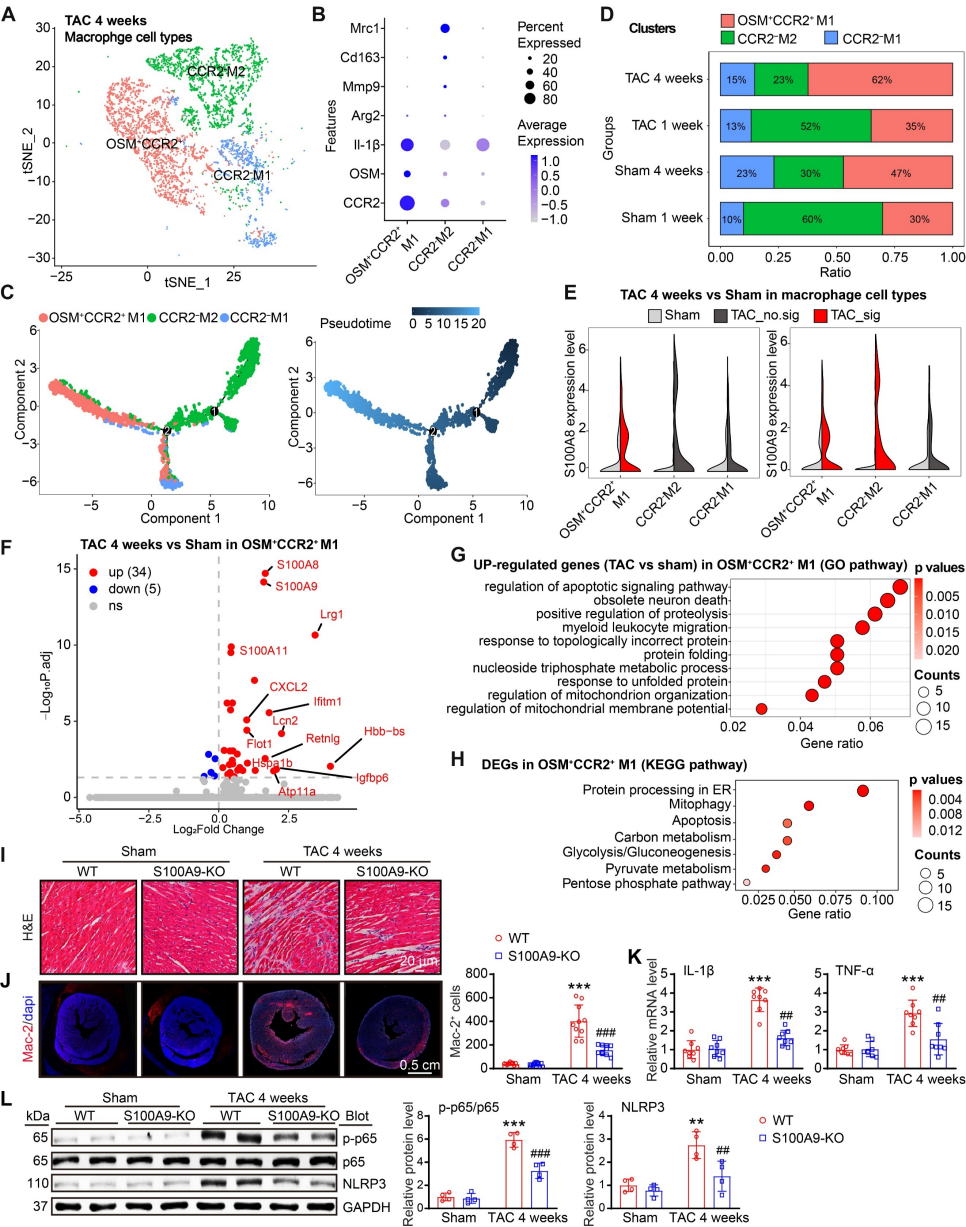
** S100A8/A9 expression is upregulated in cardiac macrophages at four weeks post TAC in mice. (A)** t-SNE plot of the three macrophage subpopulations (OSM^+^CCR2^+^M1, CCR2^-^M2, and CCR2^-^M1).** (B)** Dot plots of signature genes confirming the subpopulation identities.** (C)** Monocle analyses showing the ordering of macrophages along pseudotime trajectories. The minute dots in the illustration signify cells, with diverse colors denoting distinct clusters or states.** (D)** Percentages of the macrosubpopulations in the sham and TAC groups.** (E)** Violin plots showing the expression levels of the S100A8 and S100A9 genes in three types of neutrophils in the TAC and sham groups at 4 weeks.** (F)** Differential gene expression in the OSM2^+^CCR2^+^M1 macrophage subgroup. Volcano plots displaying all differentially expressed genes in OSM2^+^CCR2^+^ macrophages from the TAC group versus the sham group at 4 weeks. S100A8 and S100A9 were the top two genes whose expression was most significantly upregulated in the TAC group. **(G)** Gene Ontology (GO) pathway enrichment results for 300 genes upregulated in OSM2^+^CCR2^+^M1 macrophages from the TAC group compared with the sham group at 4 weeks. **(H)** KEGG pathways associated with 300 upregulated genes in OSM2^+^CCR2^+^M1 macrophages from the TAC group compared with those from the sham group at 4 weeks.** (I)** S100A9-KO and WT mice were subjected to TAC for 4 weeks to assess cardiac dysfunction and hypertrophy. Representative images of H&E-stained cardiac tissue. **(J)** Cardiac immunofluorescence staining with Mac-2 antibody (left, red) and quantification of the results (right, n = 10).** (K)** Cardiac qPCR analyses of IL-1β and TNF-α (n = 8). **(L)** Representative immunoblots of the p-p65, p65, and NLRP3 proteins, as well as the GAPDH loading control (left). The right panel shows the quantification of the results (n = 4). The values are presented as the means ± SDs (n = number of animals). **p < 0.01 and ***p < 0.001 vs. the WT + sham group; ^##^p < 0.01 and ^###^p < 0.001 vs. the WT + TAC 4-week group.

Given S100A8/A9 quickly induced the release of CCL2 and CCL6 from neutrophils in the early stage of TAC (Figure 2H-J), which may be key signals for the recruitment of CCR2^+^ myeloid cells, particularly monocytes, into injured hearts during the late stage of HF, we first assessed the effect of neutrophil S100A9-KO on Ang II-induced CCL2 and CCL6 expression and macrophage migration *in vitro*. BM-derived neutrophils were isolated from WT or S100A9-KO mice, separately, and treated with Ang II for 24 h. qPCR analysis revealed that Ang II-induced upregulation of both CCL2 and CCL6 mRNA levels in WT BM-derived neutrophils was significantly attenuated in S100A9-KO BM-derived neutrophils (Figure S5A). Further, coculture experiments of BM-derived neutrophils with macrophages revealed a marked decrease in the number of migrated macrophages cocultured with S100A9-KO neutrophils compared with macrophages cocultured with WT neutrophils after 24 h of Ang II stimulation (Figure S5B). Thus, these results confirmed that BM-derived neutrophils expressing S100A9 promote CCL2 and CCL6 secretion and subsequent macrophage migration *in vitro*. Moreover, after 4 weeks of TAC, we identified 3 distinct clusters (macrophages 1-3) in the heart on the basis of the expression of well-known marker genes (CCR2, OSM, Mrc1, and IL-1β) (Figure 4A-B), which included the CCR2^+^OSM^+^M1-infiltrated proinflammatory subset, the CCR2^-^M2 antigen-presenting (resident) subset, and the CCR2^-^M1-like phagocytic subset (Figure 4A-B) 3. Pseudotime analysis revealed that these cells experienced a transition from resident CCR2^-^M2 cells to infiltrated CCR2^+^OSM^+^M1 and CCR2^-^M1 cells at week 4 post TAC (Figure 4C).

Compared with sham treatment, TAC markedly increased the proportion of infiltrated CCR2^+^OSM^+^M1 macrophages but decreased the proportion of resident CCR2^-^M2 macrophages in the heart at 1 or 4 weeks (Figure 4D), confirming that TAC promotes the infiltration of CCR2^+^OSM^+^ macrophages possibly via the CCL2 and CCL6 chemokines. Moreover, the expression levels of both S100A8 and S100A9 were significantly increased in the CCR2^+^OSM^+^M1 subset at 4 weeks after TAC (Figure 4E), but this phenomenon was not observed at 1 week (Figure S3A). Interestingly, the S100A9 expression level was also increased in the resident CCR2^-^M2 subset 4 weeks after TAC (Figure 4E). Furthermore, the protein expression levels of S100A8 and S100A9 were increased in BM-derived macrophages in a time-dependent manner after Ang II treatment (Figure S3B). Therefore, these results suggested that S100A8/A9 expression is upregulated mainly in cardiac-infiltrated CCR2^+^OSM^+^ macrophages in the late stage of TAC-induced HF.

Next, we compared the differences in gene expression levels in CCR2^+^OSM^+^M1 macrophages in heart tissues between the TAC and sham groups and assessed their significance. A volcano plot indicated that these cells highly expressed inflammation- and fibrosis-related genes, such as S100A8, S100A9, Lrg1, S100A11, Lcn2, CXCL2, Ifitm1, and Igfbp6 (Figure 4F). GO and KEGG pathway analyses revealed that TAC-upregulated DEGs in the CCR2^+^OSM^+^M1 cluster were highly enriched in the regulation of the apoptotic signaling pathway, myeloid cell migration, protein processing in the endoplasmic reticulum (ER), mitophagy, and energy metabolism (Figure 4G-H).

To verify the effects of S100A8/A9 on cardiac inflammation through NF-κB signaling, we examined the infiltration of proinflammatory cells, the mRNA levels of proinflammatory cytokines and the protein levels of NF-κB and NLRP3 in WT and S100A9-KO mice 4 weeks after TAC. H&E and immunostaining revealed that the TAC-induced increase in the infiltration of proinflammatory cells, including Mac-2^+^ macrophages, and the mRNA levels of TNF-α and IL-1β in WT mice were dramatically reduced in S100A9-KO mice (Figure 4I-K). Moreover, we compared the protein levels of NF-κB (p-65) and NLRP3 in WT and S100A9-KO mice. Immunoblot analysis revealed that TAC-induced increases in p-p65 and NLRP3 protein levels in the heart were lower in S100A9-KO mice than in WT mice after TAC (Figure 4L). Thus, these data indicated that S100A8/A9 amplifies the TAC-induced cardiac inflammatory response after myeloid cells infiltrate the heart, possibly via activation of the NF-κB/NLRP3 pathway.

### Genetic deletion of S100A9 alleviates cardiac hypertrophy and dysfunction induced by chronic pressure overload

**Figure 5:**
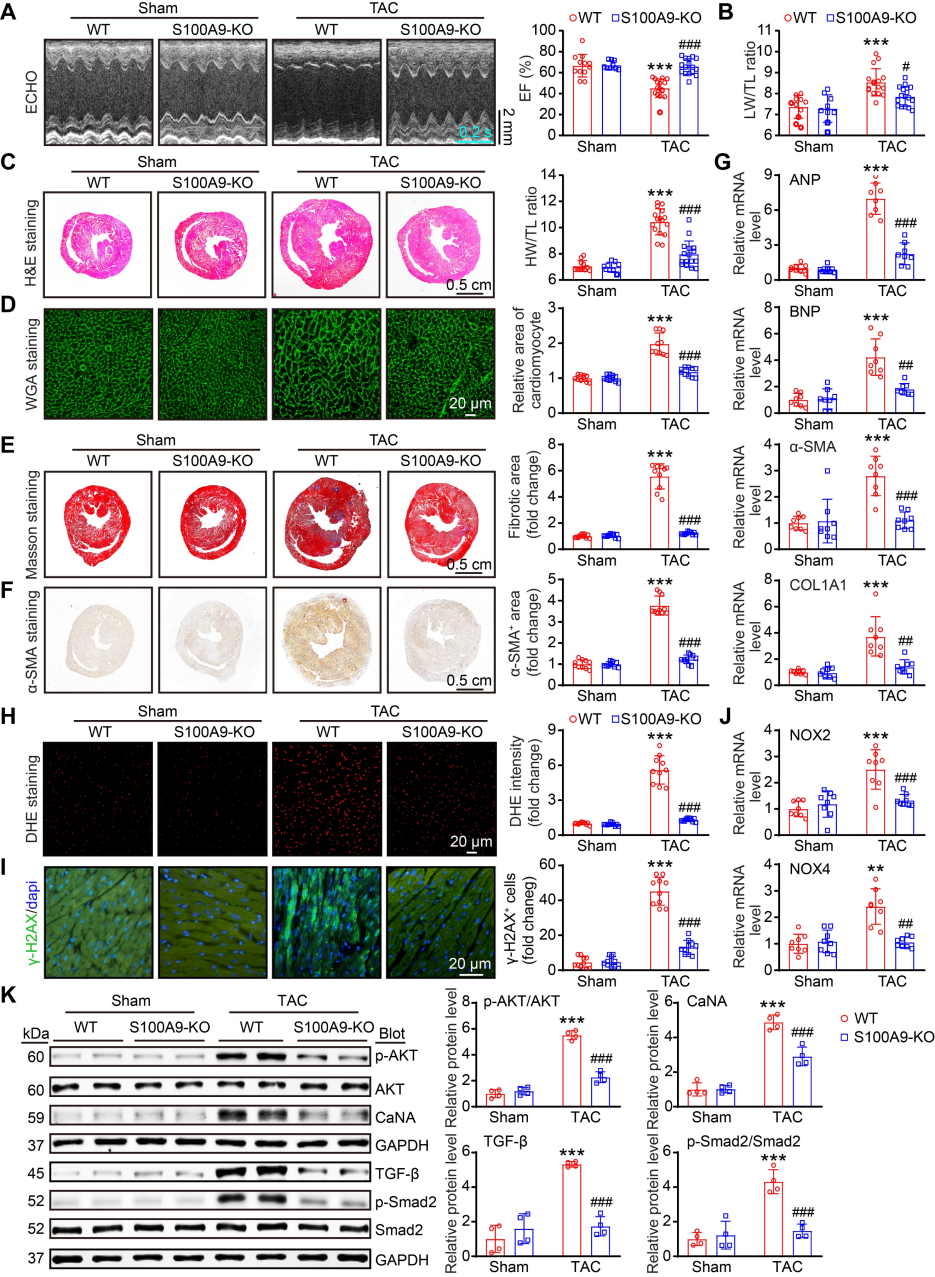
** S100A9 deficiency alleviates cardiac hypertrophy and dysfunction induced by chronic pressure overload.** S100A9-KO and WT mice were subjected to TAC for four weeks to assess cardiac dysfunction and adverse remodeling.** (A)** Representative echocardiographic images (left) and quantified EF% (right, n = 9-16). **(B)** The ratios of LW to TL (n = 9-16). **(C)** Representative images of H&E-stained cardiac tissue (left) and the HW/TL ratio (right, n = 9-16). **(D)** Representative images of cardiac tissue subjected to WGA staining (left) and the relative area of cardiomyocytes (right, n = 10).** (E)** Representative images of cardiac tissue subjected to Masson's staining (left) and the quantified fibrotic area (right, n = 10). **(F)** Cardiac immunohistochemical staining with an α-SMA antibody (left) and quantification of the results (right, n = 10). **(G)** Cardiac qPCR analyses of ANP, BNP, α-SMA and COL1A1 (n = 8). **(H)** Cardiac DHE staining (left) and quantification of the results (right, n = 10). **(I)** Cardiac immunofluorescence staining with an γ-H2AX antibody (left, green) and quantification of the results (right, n = 10).** (J)** Cardiac qPCR analysis of NOX2 and NOX4 (n = 8). **(K)** Representative immunoblots of the p-AKT, AKT, Calcineurin A (CaNA), TGF-β, p-Smad2, and total Smad2 cardiac signaling proteins, as well as the GAPDH loading control (left). The right panel shows the quantification of these proteins (n = 4). The values are presented as the means ± SDs (n = number of animals). **p < 0.01 and ***p < 0.001 vs. the WT + sham group; ^#^p < 0.05, ^##^p < 0.01 and ^###^p < 0.001 vs. the WT + TAC group.

Given that cardiac hypertrophy is one of the key mechanisms for the progression of HF 22, we investigated the effects of global S100A9 deficiency on this pathological process in WT and S100A9-KO mice. Four weeks after TAC, echocardiographic measurements revealed that the reduction in cardiac contractile function observed in WT mice, as evidenced by a reduction in the LV EF% and LW/TL ratio, was significantly improved in S100A9-KO mice (Figure 5A-B, Table S3). Moreover, compared with sham control mice, WT mice presented significant LV hypertrophy, as reflected by increased LV wall thickness, LV volume (H&E staining) and HW/TL ratio, whereas this effect was greatly attenuated in S100A9-KO mice (Figure 5C). Similarly, a reduced cardiomyocyte area was observed in S100A9-KO mice (Figure 5D). The impacts of global deletion of S100A9 on cardiac fibrosis were also corroborated by Masson's staining, immunohistochemical analysis and immunofluorescence staining. At the 4-week time point, the cardiac fibrotic, α-smooth muscle actin (α-SMA)-positive cells was significantly lower in the S100A9-KO mice than in the WT controls (Figure 5E-F). Furthermore, TAC-induced upregulation of ANP, BNP, α-SMA, and COL1A1 mRNA in WT mice was also attenuated in S100A9-KO mice (Figure 5G). Given that oxidative stress is critically involved in adverse LV hypertrophy and HF 23, we further explored whether S100A9-KO inhibits this detrimental factor in the heart. Consistently, TAC-induced increases in ROS production (DHE staining), the number of γ-H2AX-positive cells and the mRNA levels of NOX2 and NOX4 (NADPH oxidase isoforms) were lower in S100A9-KO mice than in WT controls (Figure 5H-J). Finally, we examined multiple potential pathways involved in cardiac hypertrophy and fibrosis. Immunoblot analysis revealed that the protein levels of p-AKT, Calcineurin A (CaNA), TGF-β, and p-Smad2 were markedly lower in S100A9-KO mice than in WT mice (Figure 5K). Taken together, these results suggested that the deletion of S100A9 mitigates TAC-induced cardiac hypertrophy and HF.

### Myeloid-specific deletion of S100A9 ameliorates TAC-induced cardiac hypertrophy and dysfunction *in vivo*

**Figure 6:**
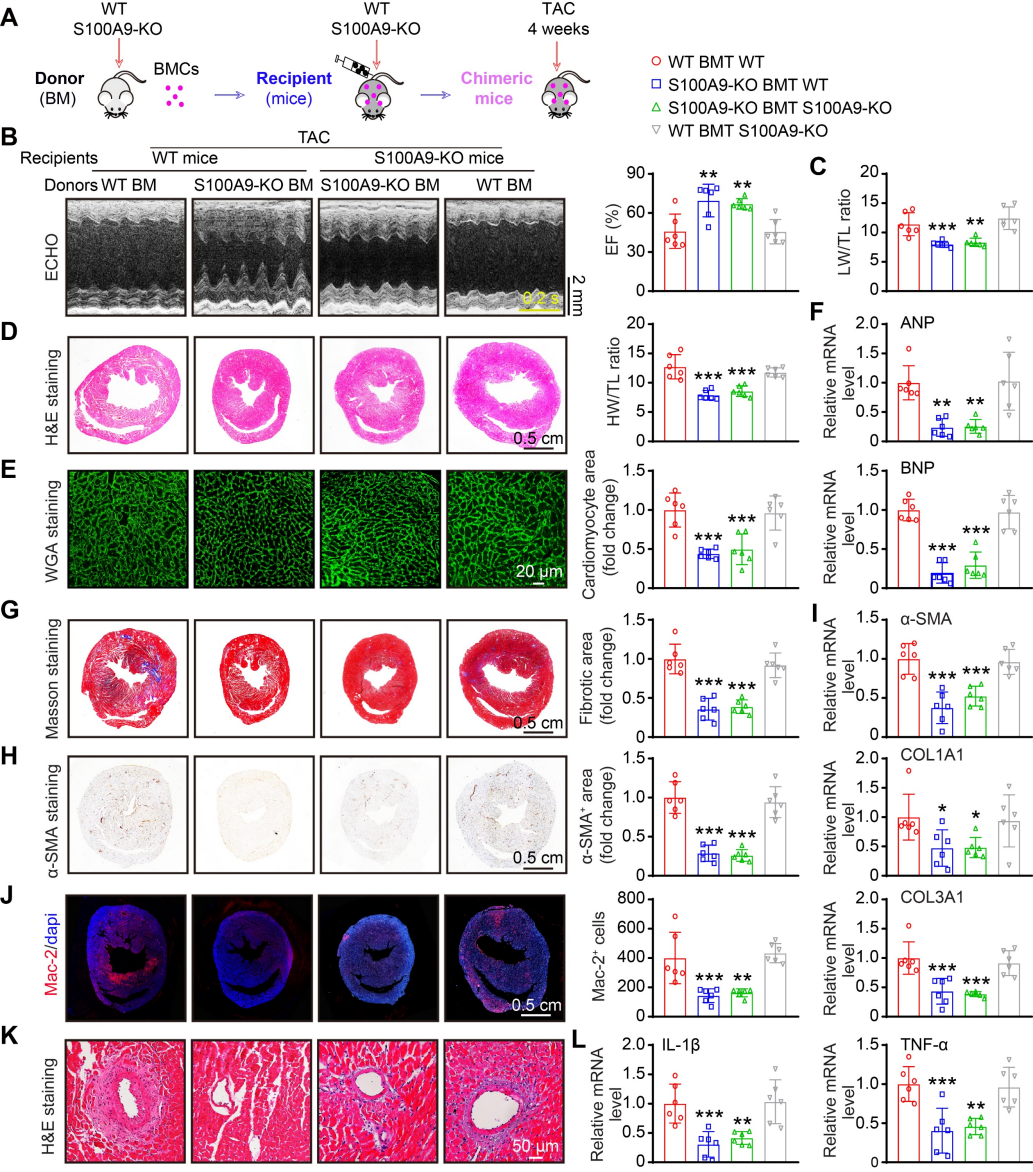
** Myeloid-specific S100A9 deletion protects against TAC-induced cardiac hypertrophy and dysfunction. (A)** Chimeric mice were generated by transplanting WT or S100A9-KO BM into WT or S100A9-KO mice for six weeks, and the mice were then subjected to TAC for four weeks to assess cardiac dysfunction and hypertrophy. **(B)** Representative echocardiographic images (left) and quantified EF% (right, n = 6). **(C)** The ratios of LW to TL (n = 6).** (D)** Representative images of H&E-stained cardiac tissue (left) and the HW/TL ratio (right, n = 6).** (E)** Representative images of cardiac tissue subjected to WGA staining (left) and the relative area of cardiomyocytes (right, n = 6).** (F)** Cardiac qPCR analyses of ANP and BNP (n = 6). **(G)** Representative images of cardiac tissue subjected to Masson's staining (left) and the quantified fibrotic area (right, n = 6).** (H)** Cardiac immunohistochemical staining with an α-SMA antibody (left) and quantification of the results (right, n = 6). **(I)** Cardiac qPCR analyses of α-SMA, COL1A1 and COL3A1 (n = 6). **(J)** Cardiac immunofluorescence staining with Mac-2 antibody (left, red) and quantification of the results (right, n = 6). **(K)** Representative stained cardiac tissue sections demonstrating inflammatory cell infiltration. **(L)** Cardiac qPCR analyses of IL-1β and TNF-α (n = 6). The values are presented as the means ± SDs (n = number of animals). *p < 0.05, **p < 0.01 and ***p < 0.001 vs. the WT BMT group.

S100A9 is expressed primarily in neutrophils and monocytes/macrophages 9, and it is highly upregulated in neutrophils and CCR2^+^-infiltrated macrophages at the early and late stages of TAC (Figure 2-3). We therefore validated whether S100A9 expression on myeloid cells is responsible for TAC-triggered cardiac hypertrophy *in vivo*. First, we generated chimeric mice by transplanting WT or S100A9-KO BM into WT or S100A9-KO mice (Figure 6A). At six weeks after transplantation, genotyping of chimeric mice performed by PCR analysis confirmed the success of BM transplantation in these mice (Figure S4A). These chimeric mice were subsequently subjected to TAC for an additional 4 weeks. The TAC-induced decrease in cardiac performance (reduced EF%) and increase in the LW/TL ratio in WT mice transplanted with WT BM were greatly attenuated in WT mice transplanted with S100A9-KO BM (Figure 6B-C, Table S4). Accordingly, 4 weeks after TAC, compared with WT mice transplanted with WT BM, WT mice transplanted with S100A9-KO BM presented noticeable alleviation of cardiac hypertrophy (decreased HW/TL ratios, myocyte areas and mRNA levels of ANP and BNP) (Figure 6D-F), myocardial fibrosis (reduced fibrotic areas, α-SMA-positive areas, and mRNA levels of α-SMA, COL1A1 and COL3A1), inflammation (decreased inflammatory cells, Mac-2-positive macrophages, and mRNA levels of IL-1β and TNF-α) and oxidative stress (reduced ROS levels, γ-H2AX-positive cells, and mRNA levels of NOX2 and NOX4) (Figure 6B-L, Figure S4B-D). These protective effects were also observed in KO mice transplanted with S100A9-KO BM (Figure 6B-L, Figure S4B-D) but were fully reversed in KO mice transplanted with WT BM (Figure 6B-L, Figure S4B-D). Collectively, these findings suggested that S100A9 in myeloid cells contributes to TAC-induced cardiac hypertrophy and dysfunction.

### Knockout of S100A9 in macrophages blocks Ang II-induced macrophage polarization, cardiomyocyte hypertrophy and fibroblast differentiation *in vitro*

**Figure 7:**
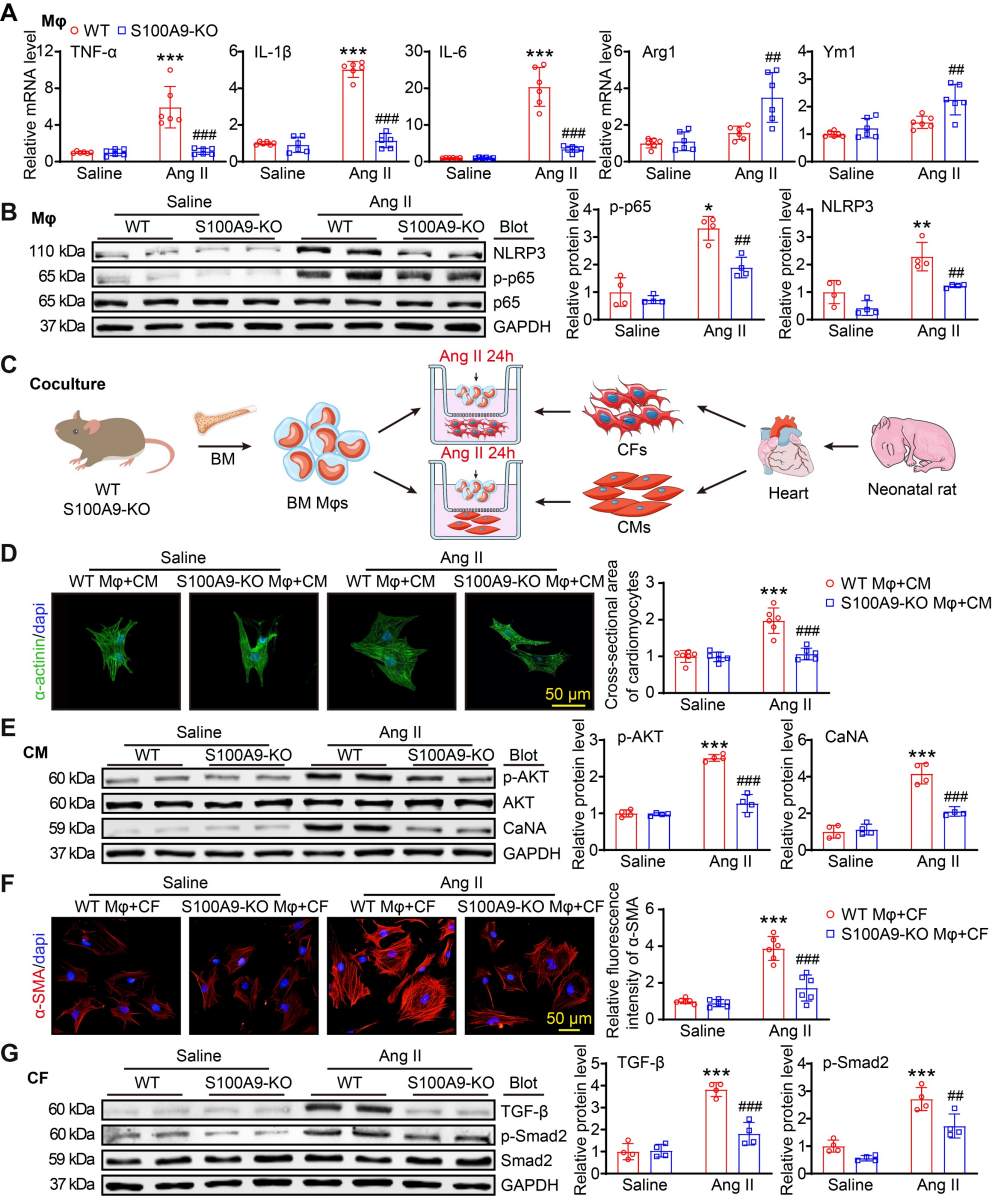
** S100A9-KO prevents Ang II-stimulated macrophage polarization, cardiomyocyte hypertrophy and fibroblast activation *in vitro.* (A)** Bone marrow (BM)-derived macrophages (Mφs) from WT or S100A9-KO mice were stimulated with saline or Ang II (100 nM, 24 h). qPCR analyses of M1 Mφ markers (TNF-α, IL-1β, and IL-6) and M2 Mφ markers (Arg1 and Ym1, n = 6). **(B)** Representative immunoblots of NLRP3, p-p65, p65 and GAPDH in Mφs (left) and quantification of these proteins (right, n = 4). **(C)** BM-derived Mφs from WT or S100A9-KO mice were cocultured with primary neonatal rat cardiac myocytes (CMs) or cardiac fibroblasts (CFs) and stimulated with saline or Ang II (100 nM) for 24 h. **(D)** Representative images of cocultured CMs immunostained with an anti-α-actinin antibody (left, green) and analysis of the relative cross-sectional area of these CMs (n = 6). **(E)** Representative immunoblots of p-AKT, AKT, CaNA and GAPDH in cocultured CMs (left) and quantification of these proteins (right, n = 4). **(F)** Representative images of cocultured CFs immunostained with an anti-α-SMA antibody (left, red) and analysis of the relative fluorescence intensity of these CFs (n = 6). **(G)** Representative immunoblots of TGF-β, p-Smad2, Smad2 and GAPDH in cocultured CFs (left) and quantification of these proteins (right, n = 4). The values are presented as the means ± SDs (n = number of replicate experiments). *p < 0.05, **p < 0.01 and ***p < 0.001 vs. the WT Mφ + saline group or vs. the WT Mφ + CM/CF + saline group; ^##^p < 0.01 and ^###^p < 0.001 vs. the WT Mφ + Ang II group or vs. the WT Mφ + CM/CF + Ang II group.

To assess whether S100A9 regulates macrophage (Mφ) function, cardiomyocyte (CM) hypertrophy and fibroblast (CF) differentiation *in vitro*, we isolated BM-derived Mφs from WT or S100A9-KO mice. After 24 h of Ang II treatment. qPCR analysis revealed that the Ang II-induced upregulation of the mRNA levels of M1 Mφ markers (TNF-α, IL-1β, and IL-6) in WT BM-derived Mφs was significantly attenuated in S100A9-KO BM-derived Mφs, and S100A9 KO promoted Ang II-induced expression of M2 Mφ markers (Arg1 and Ym1) (Figure 7A). Furthermore, immunoblot analysis of the protein levels of p-65 and NLRP3, which are crucial for Mφ polarization, revealed that the protein levels of p-p65 and NLRP3 were markedly lower in S100A9-KO BM-derived Mφs than in WT BM-derived Mφs (Figure 7B). These results suggested that the knockout of S100A9 inhibits Mφ polarization to M1 in response to Ang II stimulation.

To determine whether S100A9-KO Mφs have a direct influence on CM size and myofibroblast differentiation *in vitro*, we cocultured WT- or S100A9-KO-derived Mφs with primary neonatal rat cardiac myocytes (NRCMs) or fibroblasts (NRCFs), respectively, and stimulated them with Ang II for 24 h (Figure 7C). Immunostaining of CMs with an anti-α-actinin antibody revealed a marked increase in the NRCM size and the protein levels of p-AKT and CaNA in NRCMs cocultured with WT macrophages, whereas this effect was reduced in NRCMs cocultured with S100A9-KO macrophages (Figure 7D-E). Detection of alterations in NRCFs revealed that NRCFs cocultured with S100A9-KO macrophages expressed lower fluorescence intensities of α-SMA and protein levels of TGF-β and p-Smad2 after 24 h of Ang II treatment compared with NRCFs cocultured with WT macrophages (Figure 7F-G). Together, these results confirmed that BM-derived macrophages expressing S100A9 promote cardiomyocyte enlargement and myofibroblast differentiation *in vitro*.

### Therapeutic targeting of S100A8/A9 protects against cardiac hypertrophy and HF in a TAC mouse model

**Figure 8:**
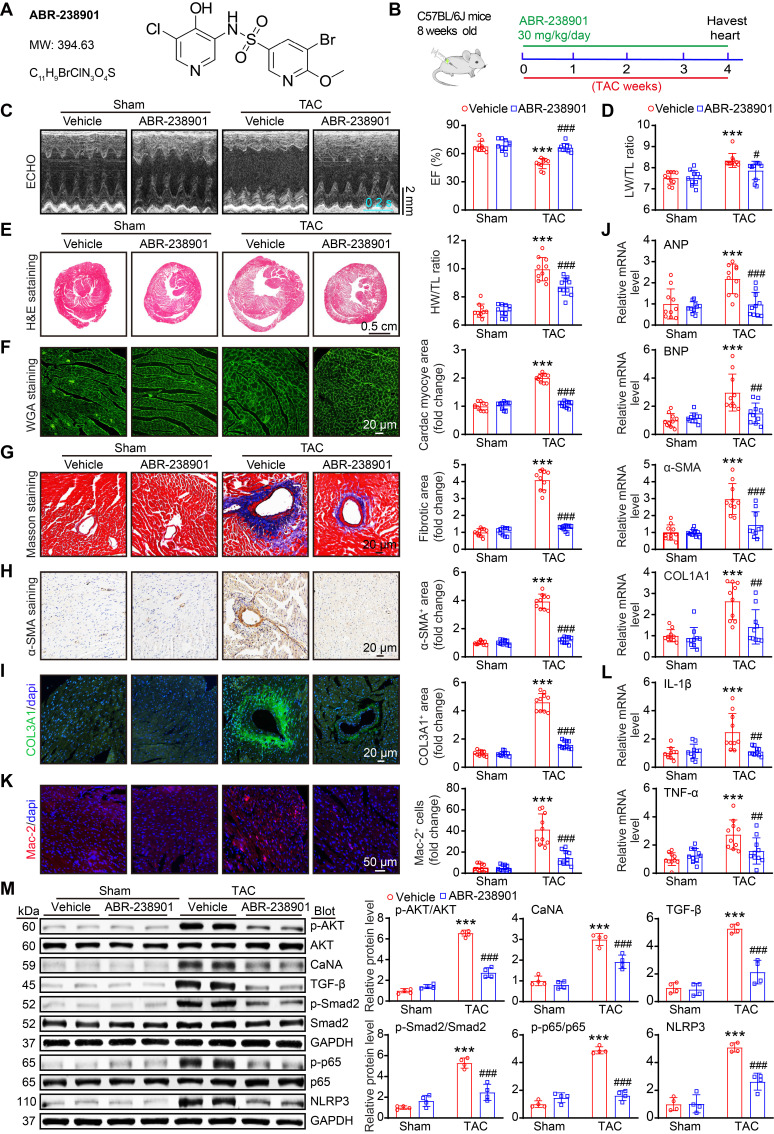
** The S100A9-specific inhibitor ABR-238901 attenuates TAC-induced cardiac hypertrophy and dysfunction. (A)** Molecular structure of the S100A9-specific antagonist ABR-238901.** (B)** WT mice were treated with ABR-238901 at a dosage of 30 mg/kg/day and subjected to TAC for 4 weeks. **(C)** Representative echocardiographic images (left) and quantified EF% (right, n = 10). **(D)** Ratio of LW to TL (n =10).** (E)** Representative images of H&E-stained cardiac tissue (left) and the HW/TL ratio (right, n = 10). **(F)** Representative images of cardiac tissue subjected to WGA staining (left) and the relative area of cardiomyocytes (right, n = 10). **(G)** Representative images of cardiac tissue subjected to Masson's staining (left) and the quantified fibrotic area (right, n = 10). **(H)** Cardiac immunohistochemical staining with an α-SMA antibody (left) and quantification of the results (right, n = 10).** (I)** Cardiac immunofluorescence staining with a COL3A1 antibody (left, green) and quantification of the results (right, n = 10). **(J)** Cardiac qPCR analyses of ANP, BNP, α-SMA and COL1A1 (n = 10). **(K)** Cardiac immunofluorescence staining with Mac-2 antibody (left, red) and quantification of the results (right, n = 10). **(L)** Cardiac qPCR analyses of IL-1β and TNF-α (n = 10). **(M)** Representative immunoblots of the p-AKT, AKT, CaNA, TGF-β, p-Smad2, Smad2, p-p65, p65, and NLRP3 proteins, as well as the GAPDH loading control (left). The right panel shows the quantification of these proteins (n = 4). The values are presented as the means ± SDs (n = number of animals). ***p < 0.001 vs. the vehicle + sham group; ^#^p < 0.05, ^##^p < 0.01 and ^###^p < 0.001 vs. the vehicle + TAC group.

Increased levels of S100A8/A9 are significantly related to the incidence of HF, and inhibition of S100A9 blocks inflammation and improves cardiac dysfunction in murine models of I/R and MI 10, 24. These findings prompted us to further determine whether the inhibition of S100A8/A9 alleviates chronic TAC-induced cardiac hypertrophy and HF in mice. WT mice were administered the S100A9-specific inhibitor ABR-238901 at a dose of 30 mg/kg in the TAC-induced hypertrophic model (Figure 8A-B). This inhibitor binds to the S100A8/A9 heterodimer and inhibits its interaction with its receptors, namely, TLR4 and RAGE 25. After four weeks, ABR-238901 treatment improved the TAC-induced decrease in EF% and the LW/TL ratio in WT mice compared with the vehicle control (Figure 8C-D, Table S5).

Moreover, ABR-238901 treatment significantly decreased the severity of cardiac hypertrophy (reduced LV wall thickness, LV volume, HW/TL ratio, and cardiac myocyte areas) and fibrosis (fibrotic areas, α-SMA-positive areas and COL3A1-positive areas), as well as decreased the mRNA expression of ANP, BNP, α-SMA, and COL1A1 in the heart, compared with the vehicle control post-TAC (Figure 8E-J). In addition, the TAC-induced increases in the infiltration of inflammatory cells (such as Mac-2-positive macrophages), ROS production, γ-H2AX-positive cells and the mRNA levels of IL-1β, TNF-α, NOX2 and NOX4 in vehicle-treated mice were markedly inhibited in ABR-238901-treated mice (Figure 8K-L, and Figure S6A-C). Additionally, the protein levels of prohypertrophic, profibrotic and proinflammatory signals, including p-AKT, CaNA, TGF-β, p-Smad2, p-p65 and NLRP3, were markedly lower in the ABR-238901-treated mice than in the vehicle-treated mice post-TAC (Figure 8M). Thus, these results demonstrated that S100A8/A9 represents a new therapeutic target for inflammation, hypertrophy and HF after chronic pressure overload.

## Discussion

**Figure 9:**
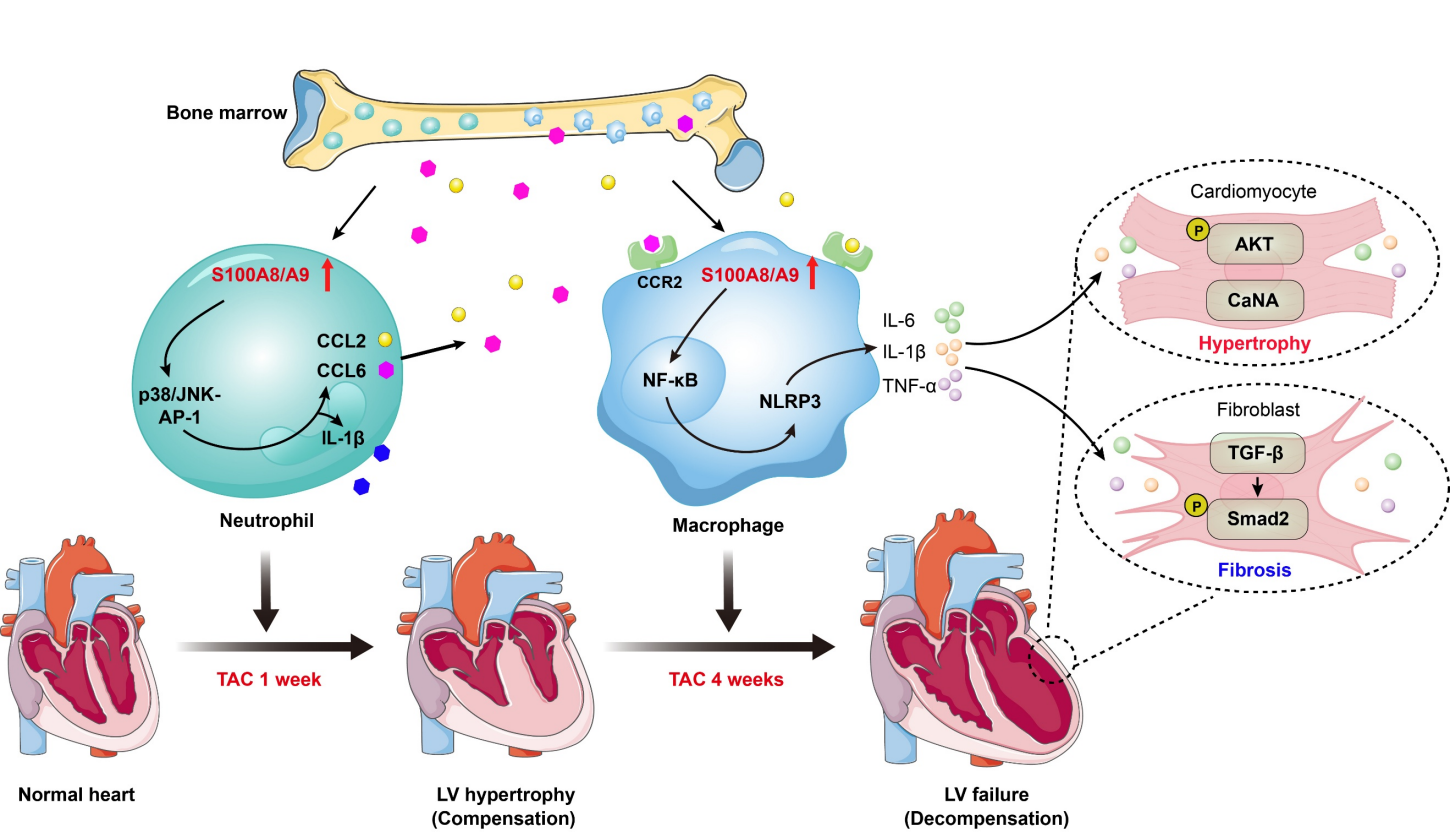
** Schematic diagram of the proposed model.** TAC induces early neutrophil infiltration, characterized by high expression of S100A8/A9. This response triggers inflammation and adaptive cardiac hypertrophy via the p38 MAPK/JNK/AP-1 axis, which induces release of CCL2 and CCL6. These secreted chemokines subsequently promote infiltration of the BM-derived CCR2^+^ macrophages, which also present elevated S100A8/A9 expression. Mechanistically, S100A8/A9 activates ER stress/NF-κB/NLRP3 signaling, which promotes the polarization of macrophages toward the M1 phenotype, while concurrently stimulating the AKT/Calcineurin A and TGF-β/Smad2/3 pathways. This cascade ultimately exacerbates maladaptive cardiac hypertrophy and accelerates HF progression.

The molecular mechanisms by which pressure overload induces the transition from compensated hypertrophy to HF are still poorly understood. In this study, using scRNA-seq analysis, we identified a significant role for S100A8/A9 in myeloid cells during the transition process induced by long-term TAC. The findings from animal models and *in vitro* experiments indicated that TAC causes early cardiac neutrophil infiltration, and the highly expression of S100A8 and S100A9 in these neutrophils triggers acute inflammation and adaptive cardiac hypertrophy possibly through p38 MAPK/JNK/AP-1-mediated production of IL-1β and CCL2 and CCL6. These chemokines subsequently promote late cardiac infiltration of CCR2^+^ macrophages that express high levels of S00A8/A9, which activate ER stress/NF-κB/Nrlp3 signaling to skew macrophages toward the M1 phenotype and promote activation of the AKT/Calcineurin A and TGF-β/Smad2/3 pathways, leading to subsequent aggravation of cardiac hypertrophy and HF (Figure 9). Thus, these findings demonstrated that in myeloid cells, S100A8/A9 induces the transition from adaptive hypertrophy to HF, suggesting that targeting S100A8/A9 is a new therapeutic strategy for the treatment of this disease.

HF is typically characterized by cardiac hypertrophy mediated by inflammation and fibrosis, and it has poor prognosis. Increasing evidence implies that the upregulation of proinflammatory cytokines and chemokines is closely related to pressure overload-induced cardiac hypertrophy and HF. S100 family members, such as S100A8 (MRP8) and S100A9 (MRP14), are Ca^2+^-binding proteins that regulating cell growth, apoptosis and the inflammatory response through binding to their receptors 11. S100A8/A9 is constitutively expressed in neutrophils and monocytes/macrophages, and its expression is strongly upregulated in response to infection, injury and stress, thereby inducing various proinflammatory cytokines and chemokines to stimulate leukocyte recruitment and subsequent organ damage 11. For example, the circulating S100A8/A9 level is highly increased in the blood of patients with sepsis 26. Moreover, during the early stages of myocardial infarction (MI) and ischemia/reperfusion (I/R), massive neutrophil infiltration occurs in the heart, leading to a rapid increase in S100A8/A9 level 10, 12. Moreover, neutrophil-derived S100A8/A9 downregulates Nrf1-NDUFA3, causing complex I deficiency and mitochondrial dysfunction, which resulting in resultant mtDNA release ZBP1-PANoptosis activation in endothelial cells 27. In addition, S100A8/A9 represents a promising diagnostic and predictive biomarker for various inflammation-associated diseases, such as sepsis and acute MI, in clinical patients 8, 9. In recent years, multiple studies have demonstrated that S100A8/A9 plays critical roles in cardiomyocyte death, mitochondrial dysfunction, and epithelial-to-mesenchymal transition (EMT) during the progression of cardiovascular diseases, such as MI, myocardial I/R injury, organ fibrosis, and diabetic nephropathy, through binding to toll-like receptor 4 (TLR4) or the receptor for advanced glycation end products (RAGE) 10-15. However, the potential role of S100A8/A9 in models of pressure overload-induced cardiac hypertrophy has received little attention. The present findings expand the knowledge of how S100A8/A9 in myeloid cells contributes to the TAC-induced transition from cardiac hypertrophy to HF and reveal that S100A8/A9 plays a critical role in the pathogenesis of HF.

Cardiac hypertrophy is an early response of the heart to pressure overload. Inflammatory cells and the production of inflammatory factors are crucially involved in this process. Neutrophils are the first immune cells to be attracted to sites of inflammation and ischemic tissues, where they are activated and produce chemokines to promote the infiltration of other immune cells, such as macrophages, in the heart 28-30. Importantly, neutrophils play critical roles in acute ischemic damage.

Depletion of neutrophils significantly reduces cardiac infarct size and dysfunction in different animal models following MI and I/R injury 28-30. Furthermore, multiple studies have revealed that neutrophils are the earliest cells that infiltrate the heart after pressure overload. After TAC, the infiltration of Ly6G^+^ neutrophils in the heart is significantly increased on Day 3, peaks on Day 7, and then decreases to baseline on Day 28, and this increase occurs earlier than monocyte infiltration 31-33. Moreover, the role of neutrophils in pressure overload-induced cardiac hypertrophy and dysfunction has been recently reported 31. Depletion of neutrophils or myeloid deficiency of Wnt5a markedly attenuates TAC-induced cardiac inflammation and hypertrophy, and it improves cardiac function in mice, suggesting that targeting neutrophils could be a new therapeutic target for HF 31. In the present study, S100A8/A9 expression was highly upregulated in neutrophils but not in macrophages after 1 week of TAC, suggesting that neutrophils are the primary inflammatory cells in the early phase of HF. S100A8/A9 markedly activated the p38 MAPK/JNK/AP-1 signaling cascade to further upregulate the expression of proinflammatory cytokines (IL-1β) and chemokines (CCL2 and CCL6), as well as increase the infiltration of other immune cells, particularly CCR2^+^ BM-derived macrophages, thereby inducing acute inflammation and adaptive cardiac hypertrophy. Conversely, S100A9 deficiency in mice not only led to marked decreases in the infiltration of proinflammatory cells, including Mac-2^+^ macrophages and Ly6G^+^ neutrophils in the heart, but also inhibited p38 MAPK/JNK/AP-1-mediated production of IL-1β, CCL2 and CCL6. These findings were consistent with previous results in tumor cells 17-21. Importantly, S100A9 deficiency alleviated TAC-induced cardiac hypertrophy and dysfunction in the early stage of TAC. Together, these findings support the hypothesis that increased expression of S100A9 in neutrophils is an early step in the initiation of inflammation and adaptive cardiac hypertrophy during TAC-induced HF.

Chronic cardiac pressure overload often elicits pathological hypertrophy and HF. Monocytes/macrophages are actively involved in inflammation during this process. Data from animal models and* in vitro* studies have demonstrated that macrophages are detected in all stages of HF and that the degree of macrophage infiltration is associated with the severity of cardiac damage 3. Pressure overload stimulation promotes the monocytes recruitment and subsequent M1-polarization of macrophages, which produces proinflammatory cytokines (TNF-α, IL-1β and IL-6) to induce the transition from cardiac hypertrophy to HF 34. Several reports have shown that ER stress activates NF-κB signaling, which increases NLRP3 inflammasome expression and IL-1β release, thereby leading to the initiation of the inflammatory response, suggesting that there is crosstalk between ER stress and the NF-κB/NLRP3 axis 35-37. Notably, these inflammatory cytokines adversely affect the phenotypes and functions of all cardiac cells by suppressing cardiomyocyte contraction, stimulating macrophage activation, and inducing myofibroblast differentiation 38, 39. Thus, it is important to elucidate the cellular heterogeneity of immune cell populations, the state of their activation, and the specific functions that trigger inflammation, hypertrophy and fibrosis during HF. Interestingly, scRNA-seq analysis has revealed the cardiac immune composition in a murine model of HF induced by TAC at 1 or 4 weeks, and analysis of cardiac CD45^+^ immune cells has revealed that the major cell subpopulations are macrophages/monocytes 3. On the basis of these previous scRNA-seq datasets, we further revealed that TAC for 4 weeks induced a substantial increase in CCR2^+^ macrophages, and high S1000A8/A9 expression shifted from neutrophils toward CCR2^+^ macrophages after 4 weeks of TAC. This shift in expression promoted the inflammatory response through the activation of ER stress/NF-κB/NLRP3 signaling, which accelerated cardiomyocyte hypertrophy and fibrosis via the activation of the AKT/Calcineurin A and TGF-β/smad2 signaling pathways. In contrast, these effects were markedly attenuated in S100A9-KO mice and WT mice engrafted with S100A9-KO BM. Overall, these findings indicated that TAC-induced infiltration of macrophages highly expressing S100A9 is critical for the induction of maladaptive cardiac hypertrophy and the progression of HF.

HF is the main cause of mortality worldwide, and heart transplantation is the most effective therapy for end-stage HF; however, the shortage of donor organs is a major obstacle in many countries 40. Drug treatments can improve symptoms and increase survival but not prevent the progression of HF. Therefore, the identification of new therapeutic targets for the treatment of this disease is urgently needed. Multiple clinical trials have suggested that anti-inflammatory therapies are beneficial for decreasing HF mortality 40. Recently, we reported that systemic short-term administration of the S100A9 inhibitor ABR-238901 in mice clearly attenuates sepsis-related cardiomyopathy, renal injury and liver injury 41-43. Moreover, the injection of mice with an S100A9 neutralizing antibody or inhibitor ABR-238901 effectively protects against cardiac damage and dysfunction after myocardial I/R and 7 days of MI 10, 24, 44, 45. However, long-term administration of ABR-238901 in mice results in a deterioration of cardiac function and left ventricle dilation, suggesting a negatively impact of S100A9 inhibition on cardiac recovery after 21 days of MI 45. In the present study, we expanded upon previous observations and demonstrated that chronic pharmacological inhibition of S100A8/A9 beneficially prevented TAC-induced pathological hypertrophy and HF in mice, suggesting that blockade of S100A8/A9 represents a new therapeutic option for HF in the clinical setting.

While promising, further investigations are essential to address the limitations for its clinical implementation. First, the exploration of the role of S100A8/A9 in cardiac hypertrophy and dysfunction was limited to a mouse model induced by TAC. Second, the potential mechanisms by which TAC and Ang II induce S100A8/A9 expression in myeloid cells are unclear. Third, additional studies are needed in female mice following pressure overload to examine the possible differences between the sexes.

In conclusion, the present study revealed a novel function of S100A8/A9 in myeloid cells in the transition from pressure overload-induced cardiac hypertrophy to HF. TAC-induced early cardiac infiltration of neutrophils highly expressing S100A8/A9 contributes acute inflammation and adaptive cardiac hypertrophy. The infiltrated CCR2^+^ macrophages expressing S00A8/A9 further amplify inflammation and aggravate the cardiac hypertrophy and dysfunction induced by chronic pressure overload. Thus, these data highlight the importance of S100A8A9 in myeloid cells during inflammation and the transition from adaptive hypertrophy to HF, and they suggest that targeting S100A8/A9 may be a promising anti-inflammatory strategy for treating this disease.

## Supplementary Material

Supplementary figures and tables.

## Figures and Tables

**Table 1:** Primer sequences

Gene	Forward primer (5'-3')	Reverse primer (5'-3')
S100A8	AAATCACCATGCCCTCTACAAG	CCCACTTTTATCACCATCGCAA
S100A9	GCACAGTTGGCAACCTTTATG	TGATTGTCCTGGTTTGTGTCC
ANP	CACAGATCTGATGGATTTCAAGA	CCTCATCTTCTACCGGCATC
BNP	GAAGGTGCTGTCCCAGATGA	CCAGCAGCTGCATCTTGAAT
COLIA1	GAGTACTGGATCGACCCTAACCA	GACGGCTGAGTAGGGAACACA
COL3A1	TCCCCTGGAATCTGTGAATC	TGAGTCGAATTGGGGAGAAT
α-SMA	TCCTGACGCTGAAGTATCCGATA	GGCCACACGAAGCTCGTTAT
CCL2	CACTCACCTGCTGCTACTCA	GCTTGGTGACAAAAACTACAGC
CCL6	TCAAGCCGGGCATCATCTTT	CTGCCCTCCTTCTCAAGCAA
IL-1β	TGCCACCTTTTGACAGTGATG	TGATGTGCTGCTGCGAGATT
IL-6	GCTACCAAACTGGATATAATCAGGA	CCAGGTAGCTATGGTACTCCAGAA
Arg1	CTCCAAGCCAAAGTCCTTAGAG	GGAGCTGTCATTAGGGACATCA
Ym1	CAGGTCTGGCAATTCTTCTGAA	GTCTTGCTCATGTGTGTAAGTGA
IL-10	CTTACTGACTGGCATGAGGATCA	GCAGCTCTAGGAGCATGTGG
TNF-α	CAGGCGGTGCCTATGTCTC	CGATCACCCCGAAGTTCAGTAG
NOX2	CTTCTTGGGTCAGCACTGGC	GCAGCAAGATCAGCATGCAG
NOX4	CTTGGTGAATGCCCTCAACT	TTCTGGGATCCTCATTCTGG
GAPDH	GGTTGTCTCCTGCGACTTCA	GGTGGTCCAGGGTTTCTTACTC

S100A8, S100 calcium binding protein A8; S100A9, S100 calcium binding protein A9; ANP, atrial natriuretic factor; BNP, brain natriuretic factor; α-SMA, α-smooth muscle actin; CCL2, C-C motif chemokine ligand 2; CCL6, C-C motif chemokine ligand 6; IL-1β, interleukin 1 beta; IL-6, interleukin 6; Arg1, arginase 1; IL-10, interleukin 10; TNF-α, tumor necrosis factor alpha; NOX2, NADPH oxidase 2; NOX4, NADPH oxidase 4; GAPDH, glyceraldehyde 3-phosphate dehydrogenase.

## Abbreviations

HF: heart failure
TAC: transverse aortic constriction
CCL2/6: C-C motif chemokine ligand 2/6
S100A9-KO: S100A9 knockout
WT: wild-type
BM: bone marrow
Mφ: macrophage
LV: left ventricular
MI: myocardial infarction
scRNA-seq: single-cell RNA-sequencing
EF: ejection fraction
BNP: B-type natriuretic peptide
DEGs: differentially expressed genes
LW/TL: lung/tibial length
HW/TL: heart weight/tibial length
HW/BW: heart weight/body weight
AW: anterior wall
PW: posterior wall
LVID: left ventricular internal dimension
COL1A1: collagen I
COL3A1: collagen III
ER: endoplasmic reticulum
α-SMA: α-smooth muscle cell
NRCMs: neonatal rat cardiac myocytes
NRCFs: neonatal rat cardiac fibroblasts
I/R: ischemia/reperfusion
EMT: epithelial to mesenchymal transition
TLR4: toll-like receptor 4
RAGE: advanced glycation end products
H&E: hematoxylin and eosin
TGF-β: transforming growth factor-β
IL-1β: interleukin 1β
IL-6: interleukin 6
IL-4: interleukin 4
OSM: oncostatin-M
Mrc1: mannose receptor c-type 1
