# Supplementary material for: Single-cell RNA sequencing reveals that myeloid S100A8/A9 is a novel regulator of the transition from adaptive hypertrophy to heart failure after pressure overload
Source: Theranostics. 2025 Jul 28;15(16):8587–608. doi: 10.7150/thno.118369 (PMC12374730; doi:10.7150/thno.118369)
Supplement: Supplementary file 1 — Supplementary figures and tables. [file thnov15p8587s1.pdf]

Supporting data

Tables

Table S1. Clinical characteristics of normal control and HF patient

Parameters	Normal (n = 119)	HF patient (n =143)	p value
Male, n (%)	57(47.89%)	84(58.74%)	0.08
Age, years	45.82±1.13	69.15±1.11	<0.001
HR, beats/min	76.27±1.11	79.10±1.55	0.423
SBP, mmHg	118.84±14.80	147.62±22.22	<0.001
DBP, mmHg	75.50±11.56	88.36±18.86	<0.001
LVEF, %	59.07±0.09	42.85±1.09	<0.001
LV diameter, mm	45.93±0.34	53.45±0.85	<0.001
LAD, mm	34.06±0.28	44.02±0.60	<0.001
IVS, mm	9.84±0.20	12.29±0.56	<0.001
LVPW, mm	9.46±0.12	10.08±0.11	<0.001
WBC, 10^9/L	6.15±0.16	6.47±0.18	0.498
SCR, µmol/L	65.41±1.25	97.45±4.20	<0.001
Total cholesterol, mmol/L	4.48±0.07	4.48±0.34	0.007
Triglycerides, mmol/L	1.29±0.06	1.35±0.11	0.253
HDL, mmol/L	1.35±0.03	1.11±0.03	<0.001
LDL, mmol/L	2.42±0.05	2.29±0.07	0.025
BNP, pg/ml	27.27±2.35	1679.98±244.75	<0.001
S100A8/A9, ng/ml	2245.58±1211.53	3783.88±1886.39	<0.001
FBG, mmol/L	4.63±0.05	6.35±0.29	<0.001

$p < 0.05$ was considered significant. HF, heart failure; HR, heart rate; SBP, systolic blood pressure; DBP, diastolic blood pressure; LVEF, left ventricular ejection fraction; IVS, Interventricular septum; LAD, left atrial diameter; LVPW, left ventricular posterior wall; WBC, white blood cell; SCR, serum creatinine; LDL, low-density lipoprotein; HDL, high-density lipoprotein; BNP, B-type natriuretic peptide; FBG, fasting blood glucose.

Table S2. Echocardiographic parameters of WT or S100A9 KO mice performed TAC operation or Sham for 1 week

Parameter	Sham		TAC 1 week	
	WT	S100A9 KO	WT	S100A9 KO
EF%	67.51 ± 2.06	66.83 ± 5.95	79.33 ± 7.45***	68.71 ± 5.26##
FS%	38.29 ± 2.62	36.39 ± 4.06	47.44 ± 7.18**	39.23 ± 3.28##
LVAW; d (mm)	1.04 ± 0.13	1.00 ± 0.10	1.36 ± 0.14***	1.16 ± 0.09#
LVAW; s (mm)	1.58 ± 0.06	1.54 ± 0.10	1.88 ± 0.14***	1.71 ± 0.14#
LVID; d (mm)	3.24 ± 0.26	3.25 ± 0.17	2.78 ± 0.48*	3.27 ± 0.25#
LVID; s (mm)	2.18 ± 0.41	2.31 ± 0.31	1.59 ± 0.38**	2.15 ± 0.32#
LVPW; d (mm)	1.01 ± 0.15	1.06 ± 0.15	1.30 ± 0.18**	1.08 ± 0.05#
LVPW; s (mm)	1.47 ± 0.15	1.39 ± 0.16	1.81 ± 0.09***	1.49 ± 0.07###

Values: means ± SD (n = 7-11);

*p<0.05, **p<0.01 and ***p<0.001 vs. WT + Sham;

#p<0.05, ##p<0.01 and ###p<0.001 vs. WT + TAC 1 week.

EF, ejection fraction; FS, fractional shortening; LVAW, left ventricular anterior wall; LVID, left ventricular internal dimension; LVPW, left ventricular posterior wall; LV Vol, left ventricular volume; d, end-diastole; s, end-systole.

Table S3. Echocardiographic parameters of WT or S100A9 KO mice performed TAC operation or Sham for 4 weeks

Parameter	Sham		TAC 4 weeks	
	WT	S100A9 KO	WT	S100A9 KO
EF%	66.66 ± 10.35	67.20 ± 3.80	45.18 ± 8.57***	65.68 ± 7.19###
FS%	36.83 ± 9.17	36.65 ± 4.09	22.24 ± 4.79***	36.01 ± 4.07###
LVAW; d (mm)	1.02 ± 0.12	1.02 ± 0.05	0.85 ± 0.14**	1.08 ± 0.16###
LVAW; s (mm)	1.53 ± 0.23	1.54 ± 0.09	1.26 ± 0.16**	1.53 ± 0.27##
LVID; d (mm)	3.25 ± 0.37	3.26 ± 0.19	3.88 ± 0.39***	3.36 ± 0.28###
LVID; s (mm)	2.07 ± 0.40	2.08 ± 0.14	2.75 ± 0.26***	2.18 ± 0.27###
LVPW; d (mm)	1.02 ± 0.16	1.04 ± 0.09	0.86 ± 0.13*	1.04 ± 0.15##
LVPW; s (mm)	1.37 ± 0.12	1.36 ± 0.10	1.15 ± 0.16**	1.33 ± 0.21#

Values: means ± SD (n = 9-16);

*p<0.05, **p<0.01 and ***p<0.001 vs. WT + Sham;

#p<0.05, ##p<0.01 and ###p<0.001 vs. WT + TAC 4 weeks.

EF, ejection fraction; FS, fractional shortening; LVAW, left ventricular anterior wall; LVID, left ventricular internal dimension; LVPW, left ventricular posterior wall; LV Vol, left ventricular volume; d, end-diastole; s, end-systole.

Table S4. Echocardiographic parameters of S100A9 BM chimeric mice performed TAC operation or Sham for 4 weeks

Parameter	TAC 4 weeks			
	WT		S100A9 KO	
	WT BM	S100A9 KO BM	S100A9 KO BM	WT BM
EF%	45.96 ±12.03	69.57 ± 11.42**	66.92 ± 3.72**	45.60 ±8.52
FS%	22.63 ±6.92	38.60 ± 8.26**	36.47 ± 4.29**	23.95 ± 4.22
LVAW; d (mm)	0.92 ± 0.16	1.19 ± 0.14**	1.19 ± 0.11**	0.94 ± 0.05
LVAW; s (mm)	1.47 ± 0.18	1.86 ± 0.19**	1.83 ± 0.06**	1.48 ± 0.18
LVID; d (mm)	4.09 ± 0.36	3.41 ± 0.33**	3.59 ± 0.18*	4.24 ± 0.21
LVID; s (mm)	3.07 ± 0.37	1.92 ± 0.29***	2.05 ± 0.36***	3.18 ± 0.37
LVPW; d (mm)	0.90 ± 0.16	1.21 ± 0.15**	1.19 ± 0.05**	0.95 ± 0.08
LVPW; s (mm)	1.45 ± 0.21	1.80 ± 0.22**	1.77 ± 0.04*	1.44 ± 0.11

Values: means ± SD (n = 6);

*p<0.05, **p<0.01 and ***p<0.001 vs. TAC 4 weeks + WT BMT WT.

EF, ejection fraction; FS, fractional shortening; LVAW, left ventricular anterior wall; LVID, left ventricular internal dimension; LVPW, left ventricular posterior wall; LV Vol, left ventricular volume; d, end-diastole; s, end-systole.

Table S5. Echocardiographic parameters of WT mice treated with vehicle or ABR-238901 and performed TAC operation or Sham for 4 weeks

Parameter	Sham		TAC 4 weeks	
	Vehicle	ABR-238901	Vehicle	ABR-238901
EF%	67.83 ± 5.23	69.00 ± 5.47	49.37 ± 5.10***	66.42 ± 4.02$^{\#\#\#}$
FS%	36.82 ± 4.30	37.83 ± 4.08	24.25 ± 2.31***	37.55 ± 5.14$^{\#\#\#}$
LVAW; d (mm)	1.04 ± 0.16	1.02 ± 0.16	0.84 ± 0.12*	1.17 ± 0.11$^{\#\#\#}$
LVAW; s (mm)	1.57 ± 0.19	1.57 ± 0.25	1.27 ± 0.09*	1.63 ± 0.19$^{\#\#}$
LVID; d (mm)	3.11 ± 0.24	3.07 ± 0.30	3.76 ± 0.17***	3.09 ± 0.24$^{\#\#\#}$
LVID; s (mm)	2.02 ± 0.22	2.07 ± 0.34	2.57 ± 0.32**	1.82 ± 0.29$^{\#\#\#}$
LVPW; d (mm)	1.00 ± 0.14	1.00 ± 0.16	0.84 ± 0.11*	1.08 ± 0.10$^{\#\#}$
LVPW; s (mm)	1.44 ± 0.19	1.35 ± 0.18	1.15 ± 0.13*	1.53 ± 0.23$^{\#\#\#}$

Values: means ± SD (n = 10);

*p<0.05, **p<0.01 and ***p<0.001 vs. Sham + Vehicle;

$^{\#\#}$p<0.01 and $^{\#\#\#}$p<0.001 vs. TAC 4 weeks + Vehicle.

EF, ejection fraction; FS, fractional shortening; LVAW, left ventricular anterior wall; LVID, left ventricular internal dimension; LVPW, left ventricular posterior wall; LV Vol, left ventricular volume; d, end-diastole; s, end-systole.

Figures

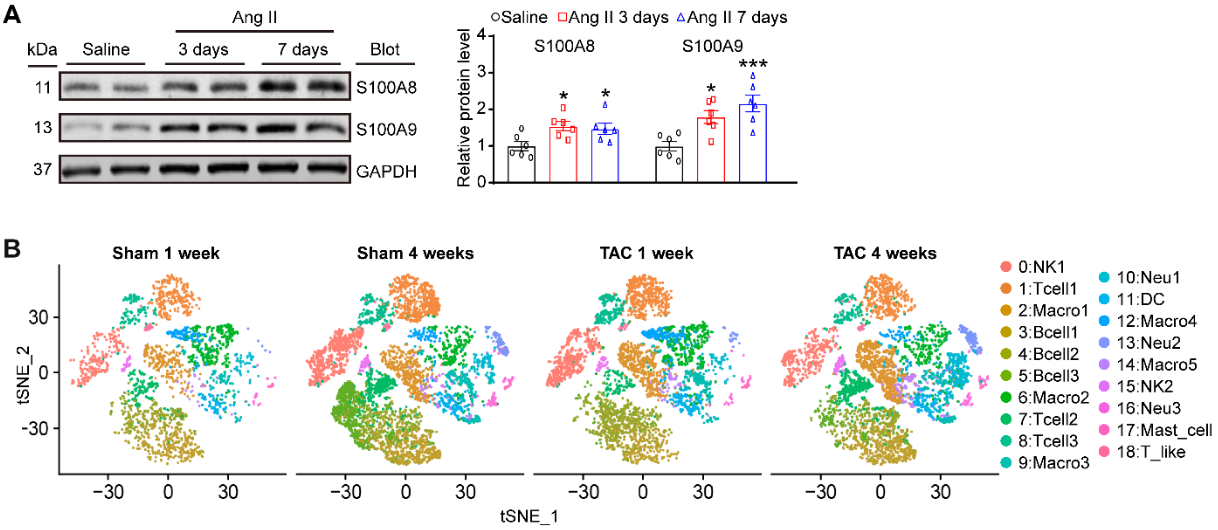

Figure S1. S100A8/A9 expression in Ang II-infused heart and cardiac subcellular localization after TAC. (A) WT mice were infused with saline or Ang II for 3 and 7 days. Representative blot of S100A8, S100A9 and GAPDH in heart (left), and quantification of these proteins (right, n = 6). **(B)** The tSNE map of $CD45^+$ cells in mouse hearts on week 1 and 4 post-TAC. Values are presented as mean ± SD (n = number of animals). *$p < 0.05$ and ***$p < 0.001$ vs. Saline group.

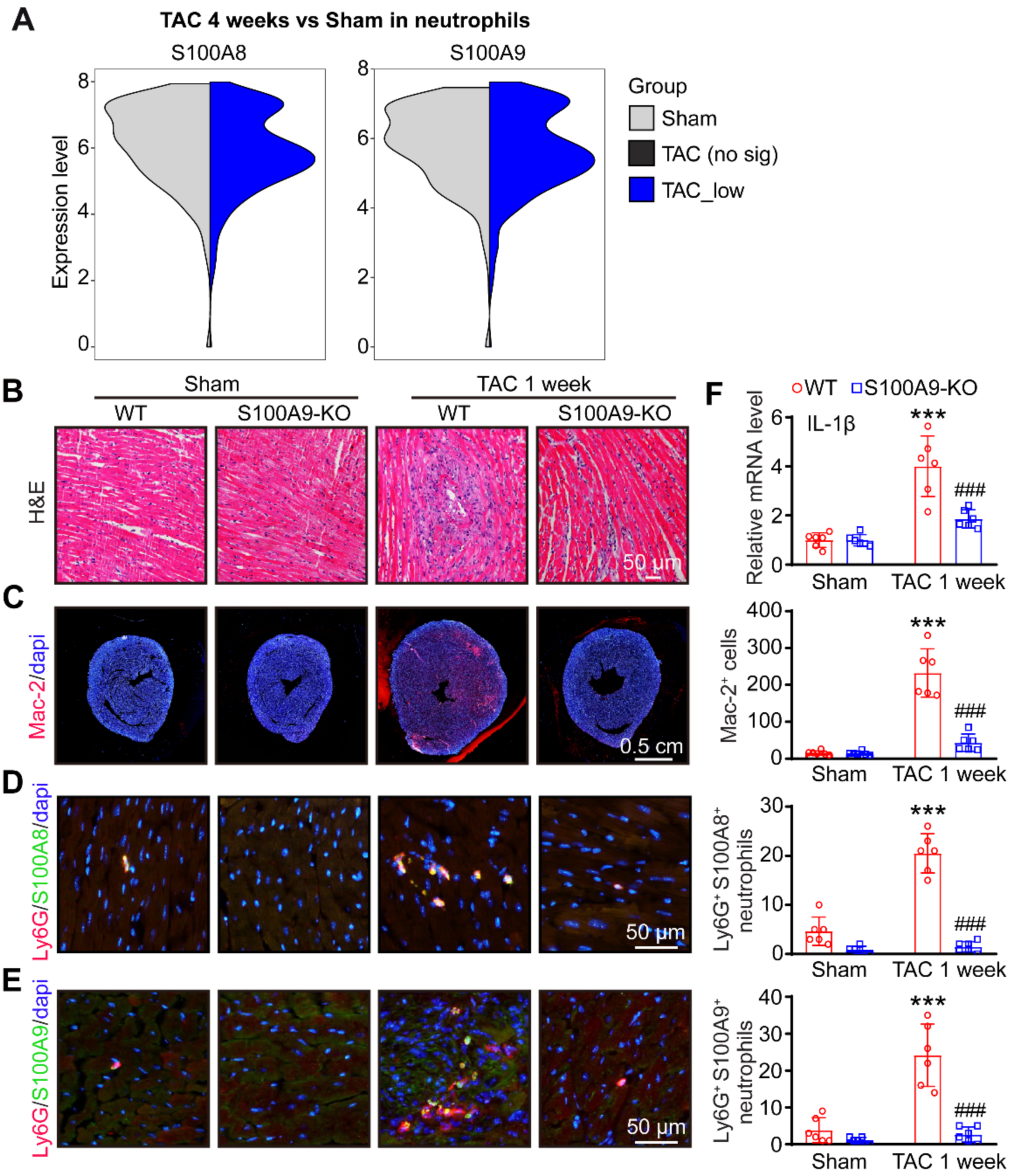

Figure S2. S100A8/A9 expression level in cardiac neutrophils after TAC. (A) ScRNA-seq analysis of S100A8/A9 expression levels in cardiac neutrophils on week 4 post-TAC. **(B)** S100A9-KO and WT mice were subjected to TAC for 1 week. Representative image of H&E-stained cardiac tissue. **(C)** Representative image of immunofluorescence staining with Mac-2 antibody (left, red) and quantification (right, n = 6). **(D)** Representative image of Ly6G (red) and S100A8 (green) immunofluorescence staining and quantification of $Ly6G^+S100A8^+$ neutrophils (right, n = 6). **(E)** Representative image of Ly6G (red) and S100A9 (green) immunofluorescence staining and quantification of $Ly6G^+S100A9^+$ neutrophils (right, n = 6). **(F)** qPCR analysis of IL-1β expression levels (n = 6). Values are presented as mean ± SD (n = number of animals). $^{***}p < 0.001$ vs. WT + Sham group; $^{###}p < 0.001$ vs. WT + TAC 1 week group.

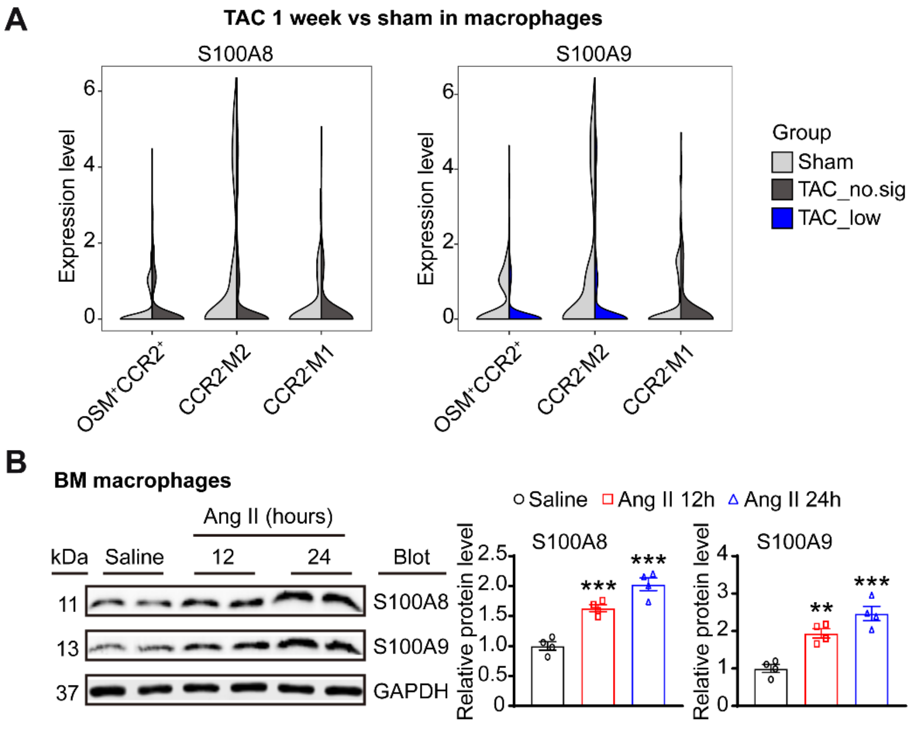

Figure S3. S100A8/A9 expression level in macrophages from TAC-induced heart or Ang II-stimulated bone marrow. (A) ScRNA-seq analysis of S100A8/A9 expression levels in cardiac macrophages on week 1 post-TAC. **(B)** Bone marrow macrophages derived from WT and S100A9-KO mice were stimulated with saline or Ang II (100 nM) for 12 and 24 hours. Representative blot of S100A8, S100A9 and GAPDH (left), and quantification of these proteins (right, n = 4). Values are presented as mean ± SD (n = number of replicate experiments). $**p < 0.01$ and $***p < 0.001$ vs. Saline group.

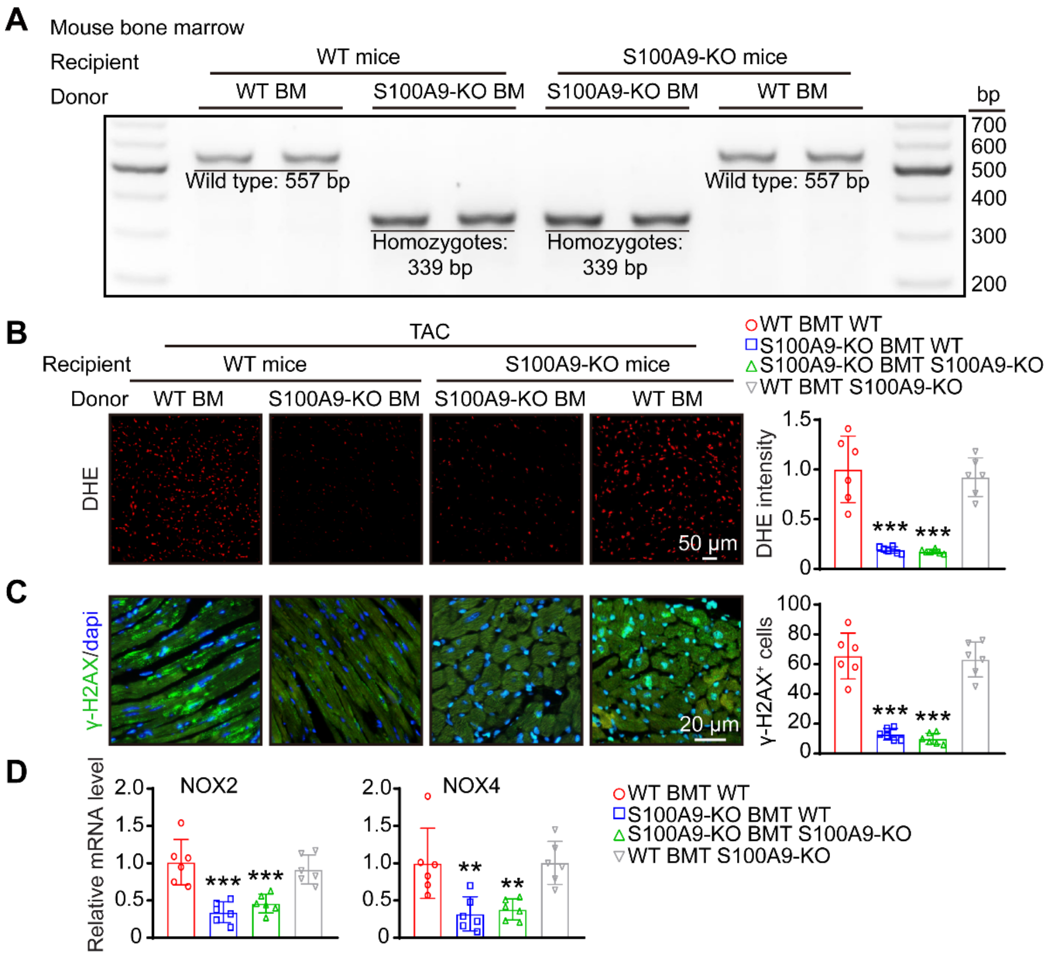

Figure S4. Myeloid-specific S100A9 deletion inhibits TAC-induced cardiac oxidative stress damage. WT or S100A9-KO mice were transplanted with BM from WT or S100A9-KO mice and subjected to TAC for additional 4 weeks. **(A)** Agarose gel electrophoresis bands for genotypic identification of mouse BM. **(B)** Representative image of DHE-stained cardiac tissue (left) and quantification of the fluorescence intensity (right, n = 6). **(C)** Representative image of cardiac immunofluorescence staining with γ-H2AX antibody (left, green) and quantification (right, n = 6). **(D)** qPCR analysis of the expression levels of NOX2 and NOX4 (n = 6). Values are presented as mean ± SD (n = number of animals). $^{**}p < 0.01$ and $^{***}p < 0.001$ vs. WT BMT WT group.

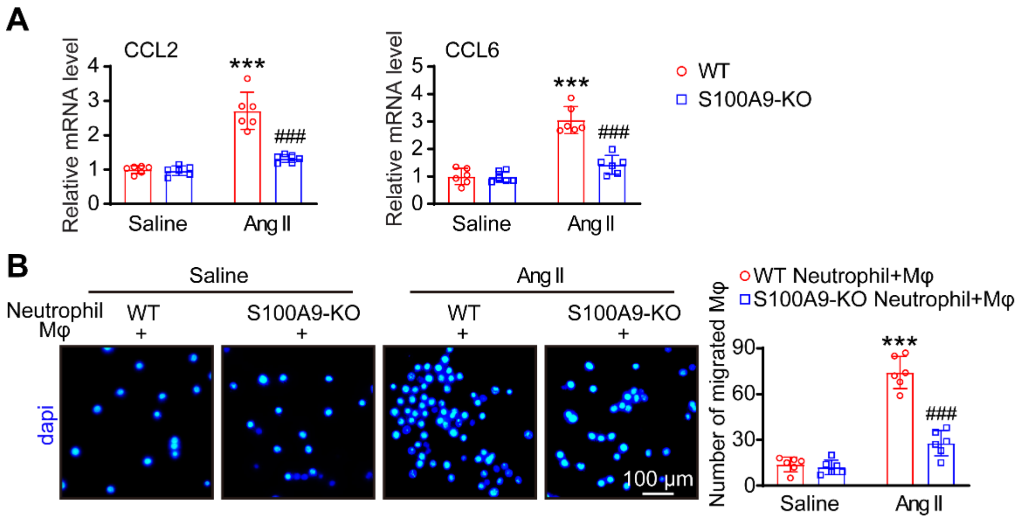

Figure S5. Knockout of S100A9 in neutrophil prevents Ang II-induced CCL2/6 secretion and macrophage migration *in vitro*. (A) Bone marrow (BM)-derived neutrophil from WT or S100A9-KO mice were stimulated with saline or Ang II (100 nM, 24 h). qPCR analyses of CCL2 and CCL6 (n = 6). **(B)** Representative images of migrated macrophage stained with DAPI (blue) and analysis of the number of migrated macrophage (n = 6).

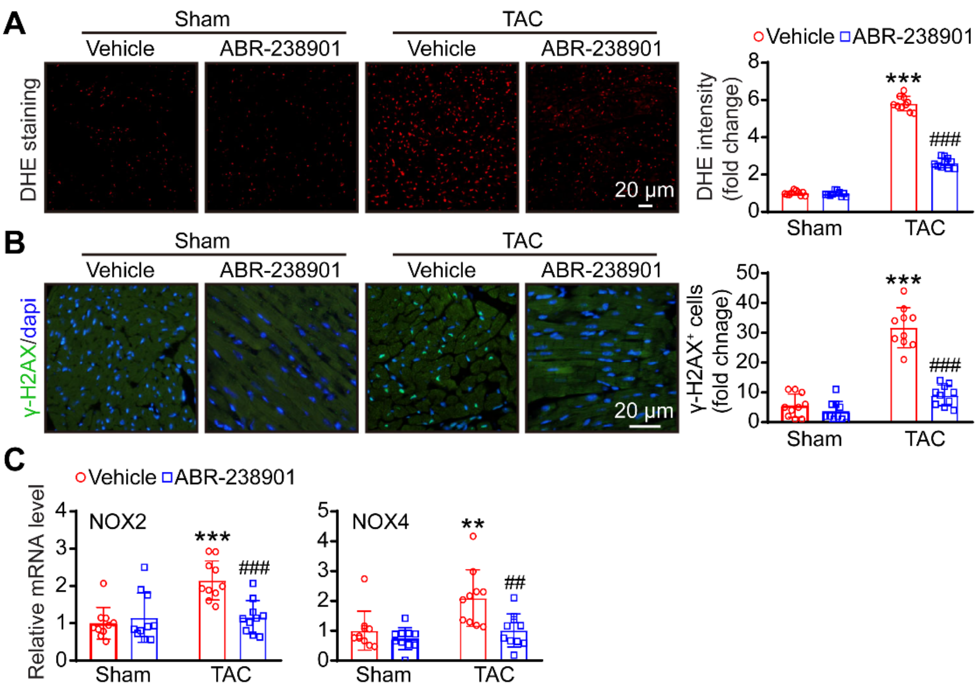

Figure S6. S100A9 specific inhibitor ABR-238901 attenuates TAC-induced cardiac oxidative stress response. WT mice were intraperitoneal injections of ABR-238901 at dose of 30 mg/kg/day for four consecutive weeks after Sham or TAC surgery. **(A)** Representative image of DHE-stained cardiac tissue (left) and quantification of the fluorescence intensity (right, n = 10). **(B)** Representative image of cardiac immunofluorescence staining with γ-H2AX antibody (left, green) and quantification (right, n = 10). **(C)** qPCR analysis of the expression levels of NOX2 and NOX4 (n = 10). Values are presented as mean ± SD (n = number of animals). **p < 0.01 and ***p < 0.001 vs. Vehicle + Sham group; $^{\#\#}$p < 0.01 and $^{\#\#\#}$p < 0.001 vs. Vehicle + TAC group.
